# Correlating Structural Properties with Electrochemical Behavior of Non-graphitizable Carbons in Na-Ion Batteries

**DOI:** 10.1021/acsaem.2c01390

**Published:** 2022-08-23

**Authors:** Blaž Tratnik, Nigel Van de Velde, Ivan Jerman, Gregor Kapun, Elena Tchernychova, Matija Tomšič, Andrej Jamnik, Boštjan Genorio, Alen Vizintin, Robert Dominko

**Affiliations:** † National Institute of Chemistry, Hajdrihova 19, Ljubljana 1000, Slovenia; ‡ Faculty of Chemistry and Chemical Technology, University of Ljubljana, Večna pot 113, Ljubljana 1000, Slovenia; § ALISTORE-European Research Institute, CNRS FR 3104 Cedex, Hub de l’Energie, Rue Baudelocque, Amiens 80039, France

**Keywords:** hard carbon, Na-ion battery, structural properties, carbonization process, SAXS, correlations, porosity, electrochemical performance

## Abstract

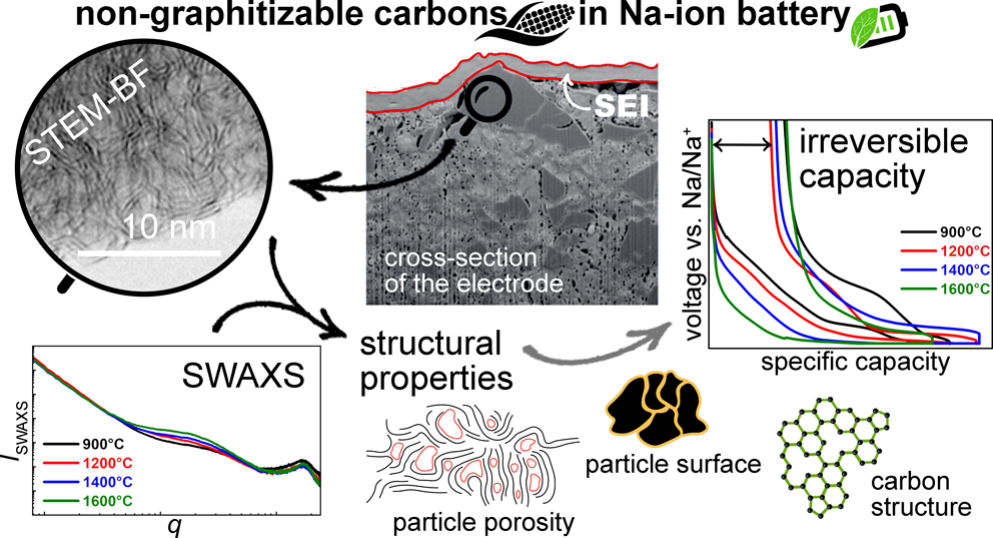

We report on a detailed structural versus electrochemical property investigation of the corncob-derived non-graphitizable carbons prepared at different carbonization temperatures using a combination of structural characterization methodology unique to this field. Non-graphitizable carbons are currently the most viable option for the negative electrode in sodium-ion batteries. However, many challenges arise from the strong dependence of the precursor’s choice and carbonization parameters on the evolution of the carbon matrix and its resulting electrochemistry. We followed structure development upon the increase in carbonization temperature with thorough structural characterization and electrochemical testing. With the increase of carbonization temperature from 900 to 1600 °C, our prepared materials exhibited a trend toward increasing structural order, an increase in the specific surface area of micropores, the development of ultramicroporosity, and an increase in conductivity. This was clearly demonstrated by a synergy of small- and wide-angle X-ray scattering, scanning transmission electron microscopy, and electron-energy loss spectroscopy techniques. Three-electrode full cell measurements confirmed incomplete desodiation of Na^+^ ions from the non-graphitizable carbons in the first cycle due to the formation of a solid–electrolyte interface and Na trapping in the pores, followed by a stable second cycle. The study of cycling stability over 100 cycles in a half-cell configuration confirmed the observed high irreversible capacity in the first cycle, which stabilized to a slow decrease afterward, with the Coulombic efficiency reaching 99% after 30 cycles and then stabilizing between 99.3 and 99.5%. Subsequently, a strong correlation between the determined structural properties and the electrochemical behavior was established.

Resource depletion and pollution related to the issues of unsustainable fossil fuel use have promoted the use of renewable and more green energy sources.^1^ To successfully integrate renewable energy resources into the electrical grid, industrial scale stationary energy storage systems are required. Secondary batteries are a promising candidate, owing to their high energy conversion and simple maintenance.^2^ Sodium-ion batteries (SIBs) are considered as the likely energy storage system to compete with lithium-ion batteries (LIBs) in large-scale applications. SIBs hold great promises due to the abundance of raw materials and homogeneous sodium distribution around the globe.^3,4^

While a number of cathode materials^5−7^ for SIBs have already been employed, the options on the anode side are more scarce. Graphite, commonly used as the negative electrode in LIBs, cannot be used in SIBs, due to its inability to form binary graphite intercalation compounds (GIC’s).^8,9^ Non-graphitizable carbons (trivially known as hard carbons) are currently the most viable option as negative electrode materials in SIBs, owing to their low price, high storage capacity, and cycling stability.^10,11^ Dahn and Stevens were the first to describe the mechanism for sodium-ion interaction with non-graphitizable carbons, by the so-called “house of cards” model following the intercalation–adsorption process.^12^ Recently, Bommier et al.^13^ proposed a different mechanism following the adsorption–intercalation–pore filling process.

However, the choice of the precursor (e.g., glucose, cellulose, lignin, and so forth) for the synthesis of the non-graphitizable carbons plays a critical role in the electrochemical mechanism and performance, due to the difference in the carbon microstructure as well as the difference in the biomass chemical composition and inorganic impurities present in the precursors.^14^ Therefore, a plethora of different biomass-derived nongraphitizable carbons with unique properties can be prepared.^15−18^ Among the biomass precursors, lignocellulosic biomass is abundant, has negative value waste, is easy to collect, and yields high carbon content.^19^ The abovementioned factors as well as the electrochemical results strongly suggest that lignocellulosic biomass is the best candidate as a non-graphitizable carbon precursor. One of the lignocellulosic waste-biomass suitable for non-graphitizable carbon is corncob. It has already been the subject of research for applications, such as supercapacitors^20−22^ as well as negative electrodes in SIBs.^23,24^

Additionally, the temperature of carbonization has an effect on the surface and bulk properties of non-graphitizable carbons. In the first step of pyrolysis, taking place at temperatures below 1000 °C, the gases that evolved throughout the temperature range define the porosity and specific surface area of carbons. During the first step, several types of porosities are formed, namely, the open, closed, and restricted porosities.^25^ Any subsequent increase of the carbonization temperature results in the growth and rearrangement of formed graphene layers, producing a more graphite-like structure. Furthermore, the rearrangement of the carbon structure at higher temperatures of carbonization critically influences the porosity and the specific surface area, arguably the two most important structural parameters to consider when designing carbon materials for application in SIBs. As a consequence of the structural rearrangement, smaller pores tend to get closed off, resulting in an increase of the closed porosity, inaccessible to different molecules. Finally, the specific surface area decreases as well.^19^ Although the exact mechanism of sodium insertion into non-graphitizable carbons is yet to be determined, the understanding of the porosity and specific surface area is crucial in establishing the correct model.

From LIBs, it has been recognized that the cycling stability is closely linked to the formation of a stable solid–electrolyte interface (SEI).^26,27^ The same is valid for SIBs. The mechanism of SEI formation depends on the formulation of the electrolyte and electrode surface properties. Additionally, the morphology of non-graphitizable carbons is susceptible to the formation of SEI. However, a clear difference between the carbon porosity and the electrode porosity should be established, as both of them influence the formation of the SEI in a different way. The carbon porosity is an outcome of intramolecular interactions. Still, not all types of carbon porosity interact with the electrolyte to form the SEI.^28^ The closed porosity is inaccessible to a number of molecules, including the electrolyte and therefore does not promote the formation of the SEI. Meanwhile, the open porosity is accessible to the electrolyte, stimulating its decomposition. On the other hand, the electrode porosity is defined as the interparticle porosity. Additional components, such as carbon additives and binders, are present in the electrode, providing an additional surface area for electrolyte decomposition. Most common electrolytes in SIBs consist of NaPF_6_ or NaClO_4_ as the salt in a mixture of various carbonate-based solvents, such as propylene carbonate (PC), ethylene carbonate (EC), and dimethyl carbonate (DMC) among others.^29,30^

In this work, we compare the structural, textural, morphological, chemical, and electrochemical properties of non-graphitizable carbons prepared from corncob in correlation with the carbonization temperature. Extensive research was dedicated to the determination of surface area and porosity of the derived materials by means of small- and wide-angle X-ray scattering (SWAXS) and transmission electron microscopy (TEM). A correlation between the evolution of the structure at different temperatures of carbonization and the electrochemical behavior was established. The SEI formation on the non-graphitizable carbon electrode was probed by focused ion beam scanning electron microscopy (FIB-SEM) and correlated with the electrochemical behavior. Finally, the feasibility of corncob-derived non-graphitizable carbons was tested in a full cell configuration.

## Results and Discussion

### Carbonization Mechanisms

Corncob was selected as the lignocellulosic biomass precursor, due to its abundance, negative bio-waste value, and the large amount of corn annually produced worldwide. This brings the corncob bio-waste into a circular economy and reduces the strain on the environment.

Lignin, cellulose, and hemicellulose, which are the main constituents of corncob, are the organic precursors that produce the best-performing non-graphitizable carbon materials.^31−33^ The complex structures of lignocellulosic biomass are composed of various compositions of the abovementioned precursors. The amount of each fraction dictates the formation of a microstructure of the biomass during pyrolysis and consequently affects the electrochemical behavior of the material. With the purpose of assessing the relative amounts of corncob constituents, thermogravimetric analysis (TGA) was performed. Pure commercial lignin and cellulose were used as reference materials.

**Figure 1:**
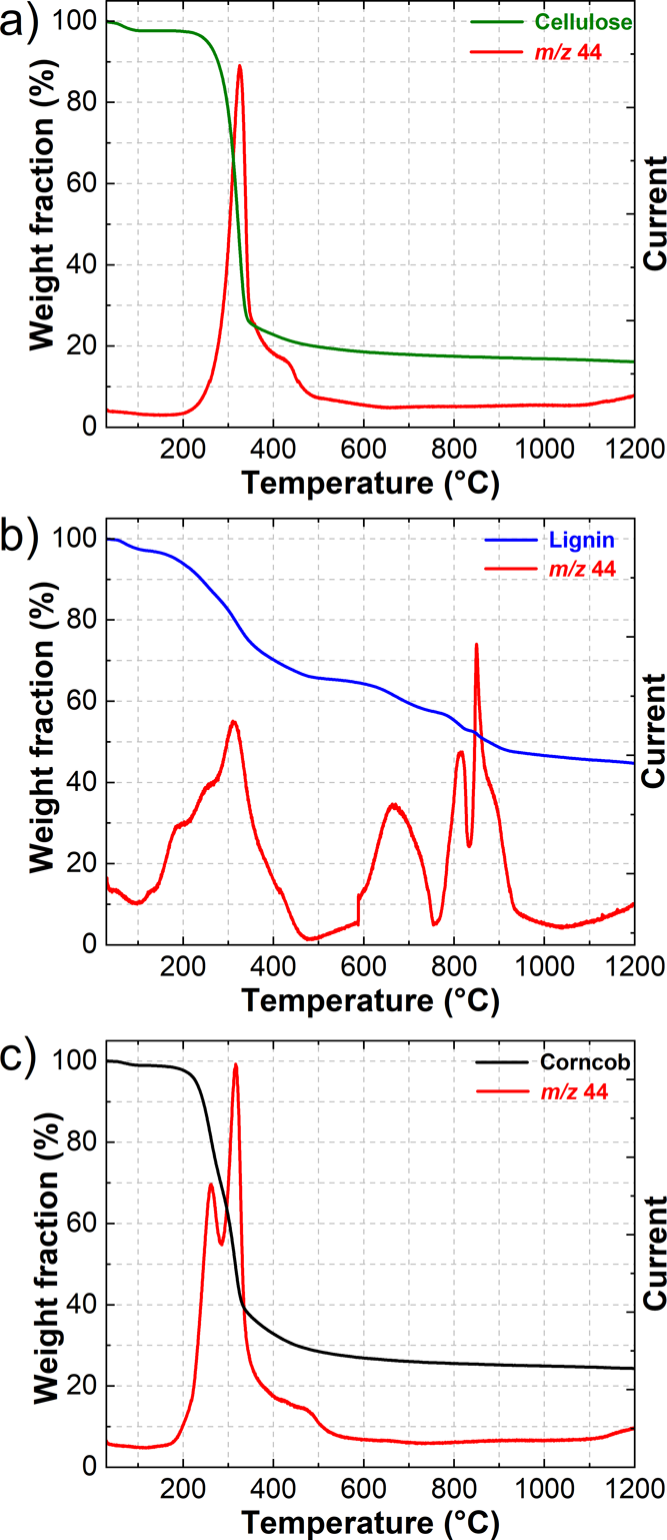
TGA coupled with mass spectroscopy of (a) cellulose reference material, (b) lignin reference material, and (c) corncob. Mass fragment *m*/*z* 44 corresponds to carbon dioxide (CO_2_).

The cellulose reference material exhibits one major decomposition step in the TG curve, taking place between 250 and 400 °C (Figure 1a). At temperatures higher than 250 °C, the glycosidic bond becomes very reactive and breaks, initiating a series of depolymerization reactions and prompting the rearrangement of the structure into monomer units such as levoglucosan and furfural. During this step, a large amount of incondensable gases is released as presented by the *m/z* 44 (CO_2_) mass fragment (Figure 1a). At temperatures above 300 °C, the conversion of pyran and furan rings occurs, followed by the formation of initial benzene rings. With increasing temperature, the concentration of the latter increases, forming the backbone of the pyrochar.^34^

In stark contrast, the lignin reference material exhibits a number of decomposition steps in the TG curve. The first major one occurs in a wide temperature range between 200 and 450 °C and is followed by several minor steps at higher temperatures (Figure 1b). In the initial stages of the first decomposition step, the conversion of the propyl chains takes place, followed by the rupture of the linkages between monomer units, resulting in a release of phenolic compounds.^34^ Additionally, at temperatures above 300 °C, the C–C bonds between alkyl chains become unstable and break, resulting in a conversion of those chains to acetaldehyde and/or acetic acid with simultaneous release of incondensable gases such as CO and CO_2_. At temperatures above 500 °C, most of the initial bonds between monomer units are broken. The reactions taking place are conversion reactions of short substituents of benzene rings. Benzene rings are very stable under an inert atmosphere; hence, the low weight loss throughout the whole decomposition process is observed. As the temperature increases, the structure of the char becomes more and more aromatic. At this point, most of the evolved compounds are incondensable gases.^34^

The TG curve of corncob resembles the one of the cellulose reference materials (Figure 1c) with only one decomposition step present. Meanwhile, the corncob retains a higher amount of weight fraction—25 wt % compared to 16 wt % for cellulose. The evolution of CO_2_ observed in the mass spectrum of cellulose (Figure 1a) demonstrates only one peak at 325 °C. In contrary, mass spectrum of corncob (Figure 1c) results in two peaks at 260 and 315 °C, indicating the conversion of multiple compounds. According to the literature, these two peaks are characteristic for the decomposition of xylan and glucomannan, the building blocks of hemicellulose. Both polysaccharides are found to decompose in two steps with a temperature shift of 20 to 30 °C with glucomannan decomposing at the higher temperature. The charring process of hemicellulose constituents is similar to that of cellulose.^34^ Therefore, the composition of corncob is presumed to comprise mainly a cellulose and hemicellulose constituent mixture and to a lesser extent lignin. At temperatures higher than 1000 °C, additional evolution of CO_2_ gas is present, indicating the start of structural rearrangement and ordering of the carbon structure. Additionally, the breaking of residual short chain functional groups from the surface is prompted, followed by the release of trapped gasses. These results are in good agreement with the results of CHNS elemental analysis (Table S1). Here, the carbon content increases with the increasing temperature of carbonization, while the content of other elements decreases.

### Structural and Morphological Properties

Structural properties, such as the interlayer spacing, the average arrangement of graphene sheets, and the overall graphitization degree of the material, were determined by XRD. The diffractogram (Figure S1) shows two characteristic peaks appearing at the values of the scattering vector of approximately 17 and 30 nm^–1^ that correspond to the (002) planes of stacked graphene sheets and (100) planes of *sp*^*2*^ hybridized carbon, respectively. The observed peaks are broad, demonstrating an absence of long-range ordering and a low degree of graphitization.

The interlayer distance, *d*_(002)_, is a crucial parameter in the characterization of non-graphitizable carbons, determining the feasibility of Na^+^ intercalation between the graphene sheets. As the temperature of carbonization increases from 900 to 1600 °C, the value of *d*_(002)_ decreases from 0.378 to 0.368 nm, respectively. All of the samples follow the trend of decreasing interlayer distance with the increasing temperature of carbonization, which is in agreement with literature reports.^28,30,35^ Interlayer distance values *d*_(002)_ determined from the fitted XRD patterns shown in Figure S1 are presented in Table 1. Despite the decreasing trend, these values are well above those of graphite (0.335 nm), favoring the intercalation of Na^+^ ions. An observed decrease of interlayer distances reflects the ongoing ordering of the carbon structure.

**Table 1:** Structural Parameters of Corncob-Derived Non-graphitizable Carbons Prepared at Different Temperatures of Carbonization^a^

	*d*_(002)_ (nm)	L_C_ (nm)	(*L*_*C*_*/d*_(002)_) +1	*I*_*D*_*/I*_*G*_	SSA N_2_ adsorption (m^2^ g^–1^)	pore volume N_2_ adsorption (10^–3^ cm^3^ g^–1^)	average pore width r_N2_ (nm)
Corn@ 900 °C	0.378(6)	0.966(5)	3.56	3.39 ± 0.07	7.3	3.5	10.0
Corn@1200 °C	0.375(9)	1.122(9)	3.99	3.15 ± 0.11	7.9	1.9	8.0
Corn@1400 °C	0.372(8)	1.176(2)	4.16	2.91 ± 0.10	8.1	1.5	8.1
Corn@1600 °C	0.367(5)	1.272(9)	4.46	2.31 ± 0.08	11.2	1.3	9.6

a *d*_(002)_ represents the interlayer distance, L_C_ is the average height of a single stack of graphene layers, (L_C_/*d*_(002)_) + 1 is the average number of graphene layers in a single stack, and *I*_*D*_/*I*_*G*_ is the concentration of defects and specific surface area (SSA) determined by N_2_ adsorption.

The effect of the final temperature of carbonization on the ordering of the structure is presented by the parameter *L*_C_, which can be calculated by eq S2 from the XRD patterns (Figure S2) and defines the height of one stack of graphene layers in the structure. The average number of graphene layers in one stack can then be calculated as [(*L*_*C*_/*d*_(002)_) + 1]. For all analyzed samples, both parameters increase with the increasing temperature of carbonization (Table 1), suggesting the formation of a graphite-like structure.

Gas adsorption measurements were performed with N_2_, and the corresponding adsorption isotherms are shown in Figure S3. All studied materials exhibit a type II isotherm characteristic of non-porous materials as well as low surface areas ranging from 7.3 to 11.2 m^2^ g^–1^ (Table 1). However, the surface area increases with increasing temperature of carbonization. This is in contradiction with the literature reports where the surface area decreases with increasing temperature of carbonization.^28,30,35^ A possible explanation for the SSA increase is the formation of additional open porosity as a consequence of gas evolution during carbonization (*m/z* 44 curve in Figure 1c). The average open pore width *r*_*N2*_, determined by N_2_ adsorption, indicates the prevalence of mesopores in the material (Table 1). The average open pore width *r*_N2*,*_ decreases from 10.0 nm for Corn@900 °C to 8.0 and 8.1 nm for Corn@1200 °C and Corn@1400 °C, respectively. However, *r*_N2_ for Corn@1600 °C increases to 9.6 nm. Yet, the total open pore volume decreases from 3.5 × 10^–3^ cm^3^ g^–1^ for Corn@900 °C to 1.3 × 10^–3^ cm^3^ g^–1^ for Corn@1600 °C. These effects most likely occur due to the growth and rearrangement of graphene-like sheets above 1000 °C, which results in sealing off of the smaller pores and formation of closed porosity inaccessible to N_2_ gas molecules. Therefore, the observed increase of SSA with the simultaneous decrease of the open pore volume can be attributed to the limitations of N_2_ gas adsorption technique. Additionally, the open pore volume decrease suggests the presence of ultramicroporosity in the investigated samples. As suggested more recently by Beda et al.,^36^ N_2_ is not suitable for assessing the smaller open pore widths in non-graphitizable carbons. Other gases, such as CO_2_, O_2_, and H_2_, should be employed when working with carbons that exhibit a high degree of ultramicroporosity.^36,37^ However, while delivering information not only on micro- and meso- but also on ultramicroporosity, the closed porosity fraction remains inaccessible even for those gas molecules.

**Figure 2:**
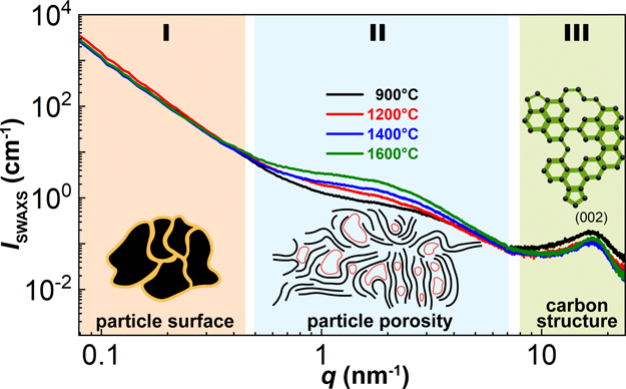
Desmeared experimental SWAXS scattering curves of corncob-derived non-graphitizable carbons. Information on different length scale dependence on the scattering vector *q* is presented.

To assess the SSA and total porosity in a higher spatial resolution, the SWAXS technique was employed. It allows probing the quantity of total porosity and offers additional insights into other important structural information, such as average pore width, micropore volume fraction, micropore surface area, and so forth (Tables S3 and S4 for complete overview). Opposite to gas adsorption techniques, SWAXS can also detect closed porosity, which in the case of Na atoms delivers additional insertion sites.^38^ Depending on different ranges of the length of scattering vector *q*, the structural information on length scales ranging from approximately 2 Å up to approximately 79 nm can be extracted from the SWAXS data. The details are encompassed by equations eqs S4–S12 and are discussed in the SI.^39,40^ The quality of the theoretical fits according to eq S4 to the experimental data is shown in Figure S4, and the resulting values of the fitting parameters are summarized in Tables S2–S4. The SWAXS patterns in Figure 2 can be divided into three regions according to the observed features.

In region I, at very small values of the scattering vector *q*, the contributions of scattering from micrometer-sized particles with well-defined interfaces to either other particles or vacuum are observed. Region II represents the scattering contributions from micropores, their clusters, and arrangements inside the carbon particles. In region III, at high values of *q*, the scattering contributions of the lamellar arrangements of the carbon atoms are detected. Although all of the contributions provided (eq S4) bestow valuable information, the contributions in region II related to the total porosity of the material (*I*_mp_) show significant differences between the materials.

From region II (Figure 2), we can deduce that the surface area provided by the total porosity of the samples, *S*_mp_, is high, ranging from 295 to 351 m^2^ g^–1^ (Table S3). The values of the calculated surface area *S*_mp_ also correlate well with the values of the total pore volume fraction, ϕ_pores_, that is, the lower total porosity corresponds to the higher surface area and vice versa (Table S3). The only exception is Corn@1600 °C in which case the value of *S*_mp_ increases instead of decreasing. The obtained values of the total pore volume fraction ϕ_pores_ from 6.1 to 8.6% increase with increasing temperature of carbonization. The estimate of the average pore width *w*_p_ (Table S3) lies in the range from approximately 0.33 to 0.52 nm. Additionally, the values of *w*_p_ follow a reverse trend compared to the values of *r*_*N2*_—it is the smallest in the case of Corn@900 °C and the biggest in the case of Corn@1600 °C. This confirms the presence of ultramicroporosity in the material, as was implied by N_2_ gas adsorption measurements. The increasing trend in the value of *w*_p_ with increasing temperature of carbonization is most likely due to the rearrangement of the carbon structure during pyrolysis, which affects the widths and lengths of the ultramicropores. Moreover, factor *f*_*a*_ is high for Corn@900 °C and Corn@1200 °C, implying a disordered structure. Subsequently, it decreases for Corn@1400 °C and Corn@1600 °C, suggesting that the pores exhibit some degree of short-range ordering.^39^

Another notable parameter determined from *I*_mp_ is the correlation length, ξ, describing the long-range order. We can observe that the value of ξ increases with the increasing temperature of carbonization, confirming previous observations that the structure of the material becomes more ordered and graphite-like. This statement is further supported by the decreasing concentration of defects (Stone–Wales defects, heteroatoms, and vacancies) with the increasing temperature of carbonization as determined by Raman spectroscopy (see Note 1 in Supporting Information and Figure S5).

Complementary to the XRD data, the wide-angle contribution, *I*_WAXS_, provides information about the evolution of the local structure of carbon on an atomistic scale. The shape of the scattering peaks in the wide-angle part of the SWAXS curves in terms of Gaussian, *w*_*G*_, and Lorentzian, *w*_*L*_, contributions was inspected. The results given in Table S4 suggest that the Gaussian contribution is negligible and the Lorentzian contribution prevails. It is known from the literature that the Lorentzian broadening is induced by the distortions in the lattice that affect long-range ordering.^39^ Therefore, the results indicate disordered nature of the studied samples, which is becoming more ordered with increasing temperature of carbonization, as inferred by the decreasing value of *w*_*L*_. Another important parameter is the fractal cut-off length, Σ, which represents the distortion length that determines the layer–layer distance above which the long-range order is lost. It is the smallest in the case of Corn@1200 °C and follows an increasing trend with increasing temperature of carbonization. In parallel, the locally flat region lengths also increase as indicated by the increasing values of the parameter *R.*

The morphologies of the corncob precursor and carbonized non-graphitizable carbons were visualized by SEM (Figure S6). The corncob precursor demonstrates a microstructure constituting fibers and voids (Figure S6a). Upon carbonization at 900 °C, the void matrix is revealed along with the macropores (Figure S6b). With increasing temperature of carbonization, the microstructure remains mainly unchanged (Figure S6c–e), similarly to the literature findings for other biomass precursors.^30,41^

**Figure 3:**
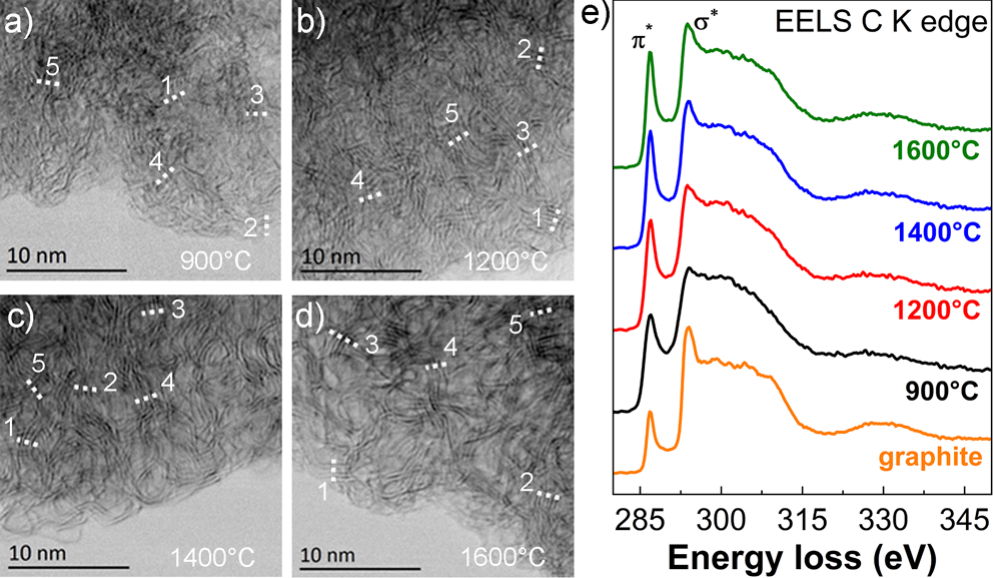
STEM-BF micrographs of pristine powder samples carbonized at (a) 900, (b) 1200, (c) 1400, and (d) 1600 °C. The dashed lines drawn at the micrographs correspond to the position of the intensity line profiles used to determine the graphite interlayer spacing (see the Supporting Information for details). (e) EELS C K edge spectra taken from all four samples as well as from the pure graphite reference sample.

Further insight into the structure of non-graphitizable carbons at the atomic level was gained via scanning transmission electron microscopy (STEM) imaging. Figure 3a–d shows the STEM-BF micrographs of all four non-graphitizable carbon samples. It can be seen, that ordering of graphene layers within the non-graphitizable carbon structure increases with the increasing temperature of carbonization. The intensity line profiles, measured at the positions marked by dashed lines (Figure S7a) demonstrate that in the case of Corn@900 °C, the arrangement of layers is rather low. For higher temperatures from 1200 °C up to 1600 °C, layers within the non-graphitizable carbon particle begin to arrange into stacks. Due to the creation of distinct stacks and their random bending and curving more pronounced ultramicroporosity can also be observed with increasing temperature.

The distances between layers in stacks are summarized in Figure S7a. Because the variations for the Corn@900 °C lie between 2.98 and 4.35 Å, average value *d* for this sample was not considered. Other samples prepared at 1200, 1400, and 1600 °C are found to have *d*-spacings equal to 3.28 ± 0.36, 3.51 ± 0.11, and 3.37 ± 0.1 Å, respectively. The measured *d* values, however, are smaller than those determined from the XRD analysis (3.76, 3.73, and 3.68 Å, respectively). This is mostly due to the assessed region, which is smaller in STEM imaging, compared to the XRD technique (30 × 30 nm vs 10 × 10 mm, respectively) and can potentially affect the determination of average values.

To further probe changes in the electronic structure of the material upon thermal treatment, STEM-electron-energy loss spectroscopy (EELS) C K edge spectra were taken from all four samples and compared to the commercially available graphite powder (Figures 3e and S7b). All spectra were deconvoluted to remove the plural scattering, background-subtracted, and normalized. The main features observed in the C K edge spectra are a sharp and defined peak at 285 eV induced by the *1s* to π* state transitions (*sp^2^* C=C double bonds), followed by a series of peaks above 290 eV due to the *1s* to σ* state transitions (*sp*^*3*^ C–C single bonds). With the increasing temperature of carbonization, the definition and intensity of the π* peak increases. In the feature at around 291 eV, the σ* peak also increases linearly with the increasing temperature, resembling that of the reference graphite spectrum (Figure 3e and S7b, orange spectrum). Both changes in the electronic structure are attributed to the increase in the crystallinity, that is, an improvement of the periodicity and symmetries of atomic positions, as well as to the increase of the long-range order with the higher temperatures of carbonization.^42−44^

The *sp^2^*/*sp^3^* ratios calculated according to the method described by Berger et al.^42^ and Daniels et al.^43^ were found to be 4.12 for Corn@900 °C; 4.71 for Corn@1200 °C; 5.17 for Corn@1400 °C; and 5.36 for Corn@1600 °C (Table S5). This demonstrates a clear increase in *sp^2^* content with the increase of carbonization temperature. For the purpose of comparison, assigning the *sp^2^*/*sp^3^* ratio to be 100% for Corn@1600 °C, the relative *sp*^*2*^ amounts of other investigated samples would be equal to 77, 88, and 97% for Corn@900 °C, Corn@1200 °C, and Corn@1400 °C, respectively (Table S5). Additionally, the *sp^2^*/*sp*^*3*^ ratio is an intrinsic property of the prepared non-graphitizable carbons and is connected to its electronic conductivity. Therefore, the increase in the relative *sp^2^* amounts with the temperature also increases the electronic conductivity of the non-graphitizable carbons. The results concur with the observation of larger number of arranged stacks of graphene layers seen by the high-resolution STEM image analysis (Figure 3a–d). There, the structural difference between Corn@1400 °C and Corn@1600 °C is much less pronounced. Compared to the graphite reference, an additional intensity in the dip between π* and σ* peaks at 287–289 eV region can be seen in all spectra, being the highest in Corn@900 °C (Figure S7b). It is ascribed to the presence of C–H (σ*) antibonding orbital^45^ due to the presence of residual hydrogen in samples, which is absent in pure crystalline graphite. The feature reduces only slightly with the increase of carbonization temperature up to 1600 °C.

All of the techniques presented above contributed a piece of information on the structural, textural, and morphological properties of the prepared corncob-derived carbons. Combining the results of all of the applied methods, a clear trend could be observed. The sample prepared at the lowest temperature exhibited the least ordering of graphene layers as well as the highest concentration of structural defects. With the increase in the carbonization temperature, a gradual rearrangement of the materials’ structure and further stacking of the graphene layers along with simultaneous formation of ultramicroporosity was observed. Such changes in the morphology and structure are expected to translate into the specific electrochemical behavior.

### Electrochemical Characterization

**Figure 4:**
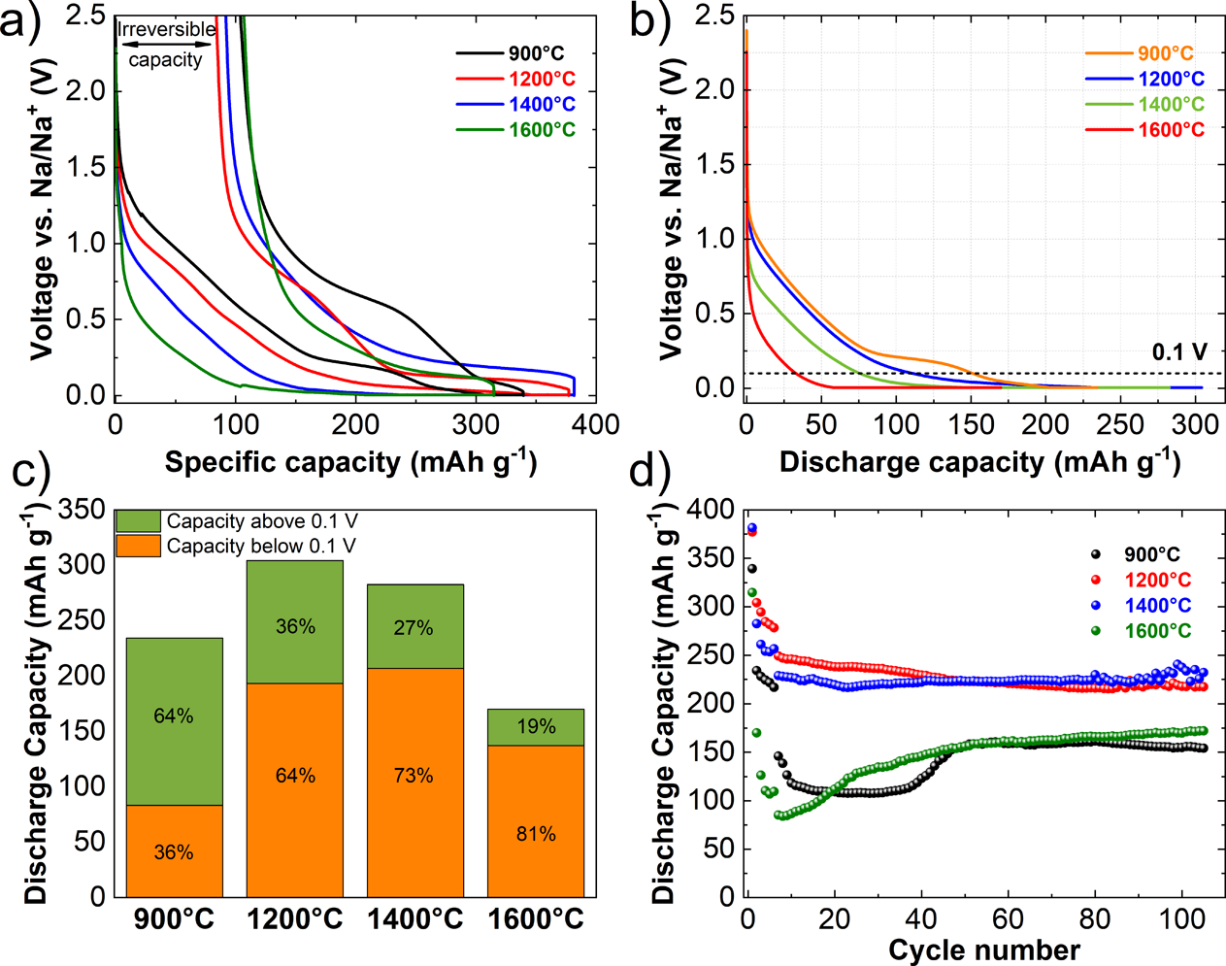
a) Galvanostatic curves in the first cycle of corncob-derived non-graphitizable carbons carbonized at different temperatures, (b) galvanostatic curve in the second discharge, (c) capacity contributions of sloping and plateau regions, and (d) cycling stability over 100 cycles.

Initial Coulombic efficiency (iCE) is one of the most important parameters to consider when determining the feasibility of the negative electrode material for Na-ion batteries. The value of the iCE dictates the loss of the active material in the first cycle due to the electrolyte decomposition and SEI formation. It is well accepted that a high surface area consumes a larger amount of charge, consequently resulting in a lower iCE.^28^ The iCE values of corncob-derived non-graphitizable carbons are calculated from the data shown in Figure 4a and are listed in Table S6 in the SI. The highest iCE value is achieved with Corn@1200 °C and amounts to 77.8%, while Corn@1600 °C exhibited the lowest iCE value of 66.1%. Taking into account, the N_2_ gas adsorption-derived SSA, we notice the same trend as reported in the literature^46^—the higher SSA_N2_ induces a higher irreversible capacity resulting in lower iCE values as is evident in the case of Corn@1600 °C. However, the differences in SSA_N2_ are minimal and smaller fluctuations should be expected in iCE’s of individual samples. Meanwhile, SWAXS results establish a clear correlation between the surface area of micropores (*S*_mp_) (Table S3) and the iCE—higher *S*_mp_ results in a lower iCE. However, SWAXS measurements comprise both open and closed porosities, and while we cannot quantify the amount of closed porosity we also cannot be certain that the iCE values refer to the total porosity.

There are several additional parameters influencing the iCE’s that have to be considered. As shown by Beda et al.,^28^ the surface area of ultramicropores (CO_2_ adsorption), the amount of active sites, and the amount of oxygen-based functional groups all heavily influence the iCE’s. Still, other techniques such as Raman spectroscopy (Note 1 in the Supporting Information), elemental analysis (Table S1), and EELS (Table S5) suggest that the amount of active sites and oxygen-based functional groups decreases with the increasing temperature of carbonization. According to this, we should expect an increase of iCE with increasing temperature of carbonization, yet that is not the case. Another phenomenon we have not considered yet is the sluggish Na^+^-ion kinetics. With the increase of the graphitization degree, it becomes harder for Na^+^ ions to intercalate between the graphene sheets. The same applies when the process of deintercalation is taking place. Also taking into account the curvature of the graphene layers in non-graphitizable carbons, it is possible that the Na^+^ ions are unable to deintercalate from the carbon structure, resulting in the loss of active material. While the increased number of defects dictates the iCE at lower temperatures of carbonization (Corn@900 °C), it appears that the sluggish Na^+^-ion kinetics is the main reason for low iCEs in the case of higher carbonization temperatures (Corn@1600 °C).

The temperature of carbonization plays a major role in the evolution of carbon structures, affecting the electrochemical behavior of prepared carbons. This effect can be distinguished within the two different regions of Na^+^ insertion into the non-graphitizable carbons, namely, the sloping region above 0.1 V and the plateau region below 0.1 V.^13^ The second discharge galvanostatic curves of the investigated carbons are presented in Figure 4b. The amount of the capacity contributed by the sloping region, which is attributed to the adsorption of Na^+^ ions onto the defects is much more significant at lower temperatures of carbonization as was already predicted by the defect concentration from Raman (Note 1 in the Supporting Information) and the SWAXS parameter *w*_L_ (Table S4). Quantitatively speaking, the sloping region amounts to 64% of the overall capacity (Figure 4c) for Corn@900 °C. As the temperature of carbonization increases, the sloping region contribution starts to diminish, while the plateau region—attributed to the intercalation of Na^+^ ions and pore filling of Na atoms—increases. For Corn@1600 °C, the sloping region contributes merely 19%, while the plateau region contributes an overwhelming 81%. This is in good agreement with structural characterization results. Several parameters obtained from SWAXS results, that is, the correlation length beyond which the long-range ordering is lost, ξ, the length beyond which the regions cannot be considered locally flat, *R*, and the distortion length which determines the layer–layer distance above which the long-range order is lost, Σ, gathered in Table S4 correlate well with the results on the capacity contribution of the plateau region. These parameters confirm the ordering of the non-graphitizable carbon structure with increasing temperature of carbonization. Specifically, as the structure gets more graphite-like, the intercalation process (Figure 4a–d) becomes more favorable than the adsorption of Na^+^ ions. This can also be observed from the electrochemical curves, as shown in Figure 4b. The sloping region transitions to the plateau region much earlier as the temperature of carbonization increases. Additionally, the voltage hold at a lower cut-off voltage of 5 mV contributes to a considerable amount of capacity. The reason for this lies in the bigger size of Na^+^ ions compared to Li^+^ ions, inducing transport and diffusion limitations.^46^ As the voltage hold begins (Figure S8a), the current begins to fall, providing favorable conditions for more facile intercalation of Na^+^ into the structure of non-graphitizable carbons. The intercalation of Na^+^ proceeds until the given conditions are met. The voltage hold step is necessary when working with non-graphitizing carbons so that the maximum capacity can be extracted from the material.^32^ Additionally, at very low sodiation potentials (in our case, 5 mV), there is a possibility of Na metal deposition on the carbon surface. According to the literature, Na deposition is designated by an overpotential that shows as a minimum in the negative voltage regime of the galvanostatic curve. The minimum is followed by an increase in voltage while still remaining negative. This increase is designated as a plateau, indicating the nucleation of sodium on the surface of the carbon electrode.^47,48^ To exclude the possibility of Na metal deposition during the sodiation at low potentials, electrochemical measurements with sodiation to negative potentials were performed. As presented in Figures S8a,c, galvanostatic profiles resembling the Na metal deposition were observed. At slower rates (C/10), the minimum is not that prominent and slowly shifts into a plateau. Meanwhile, at higher rates (1C), the minimum is observed as a sharp peak, followed by a plateau. The minimum occurs close to −0.2 V in both cases. While these results quite clearly present the deposition of Na metal, no such phenomenon is observed in the potential range around 0 V, indicating that no Na metal deposition occurs in the plateau region. Moreover, no such phenomenon is observed throughout the cycling process of our non-graphitizable carbons discharged to 5 mV.

Discharge capacity during cycling is shown in Figure 4d. Rapid fall of high initial capacities is observed for all samples in the initial five formation cycles at a current density of C/10. After switching to a higher current density of 1C, a further decrease of capacity is observed before the cycling stabilizes. The Corn@1200 °C and Corn@1400 °C samples exhibited the best performance, reaching 218 and 232 mAh g^–1^, respectively, at the end of cycling at 1C current density. The Corn@900 °C and Corn@1600 °C samples, on the other hand, demonstrated a much worse electrochemical performance.

An extensive characterization of structural, textural, and morphological properties was performed to determine the main factors influencing the electrochemical performance of corncob-derived non-graphitizable carbons. Based on the obtained results, correlations were established to assign the processes taking place either in the sloping or in the plateau region. Figure S9 presents the correlations established for the Na storage contributions in the sloping region. Despite the various parameters obtained, only the concentration of defects (*I*_D_/*I*_G_ ratio) correlated well with the relative sloping capacity. We could observe that Corn@900 °C exhibits the highest relative sloping capacity, while also exhibiting the highest concentration of defects. For other carbons, the concentration of defects begins to decrease with increasing temperature of carbonization. Concurrently, the relative sloping capacity starts decreasing as well, reaching the lowest value for Corn@1600 °C. This indicates the predominant effect of the adsorption of Na^+^ atoms on the defect sites in the carbon structure at higher potentials. Meanwhile, a considerable amount of factors influence the plateau region (Figure S10a). Parameters determined by SWAXS, such as the correlation length describing the long-range order (ξ) and the length of locally flat regions (*R*), show a strong correlation with the plateau region contributions (Figure S10b). Both parameters implicate that the increased ordering of the structure increases the relative plateau capacity. Correlation length describing the long-range order increases with the increasing temperature of carbonization, forming favorable conditions for the intercalation of Na^+^ ions into the graphite-like structure of non-graphitizable carbons. Additionally, the increasing length of locally flat regions decreases the curvature of the graphene layers, further alleviating the insertion of Na^+^ ions into the structure. However, not only does intercalation take place in the plateau region but also additional parameters related to the porosity of the material correlate well with the plateau region contributions. In this regard, the average pore width (*W*_p_) and the pore volume fraction (Φ_p_) imply that the pore filling mechanism also takes place in the plateau region (Figure S10c). We can see that with the increasing pore width and pore volume fraction, the relative plateau capacity also increases. This is a clear indication that the filling of the pores with Na atoms occurs in the low-voltage region. However, with the techniques used, we were not able to determine during which part of the plateau each of the processes (intercalation and pore filling) takes place. In summary, the concentration of defects play a decisive role in the sloping region, while a combination of graphite-like domains and the pore architecture dictate the Na storage in the plateau region.

**Figure 5:**
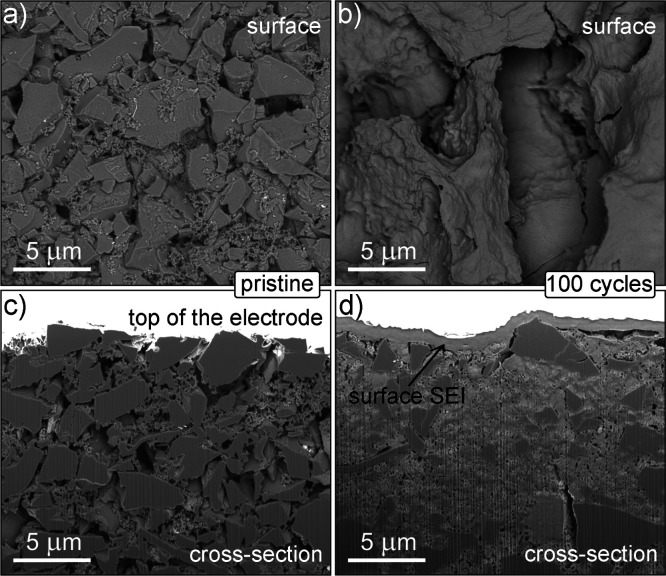
Phase contrast images of sample surfaces—top-down view: (a) pristine, (b) cycled, and (c,d) the corresponding FIB-polished cross sections of (c) pristine and (d) cycled.

To evaluate the cycling stability over 100 cycles and to see the differences between the pristine Corn@1400 °C electrode and Corn@1400 °C electrode cycled for 100 cycles, we performed a postmortem morphological analysis by FIB-SEM. The Corn@1400 °C was chosen due to the best electrochemical performance. The resulting phase contrast images of Corn@1400 °C sample surfaces are depicted in Figure 5. The top-down phase-contrast SEM image in Figure 5a shows a surface of the pristine Corn@1400 °C electrode comprised active material particles (size ≤10 μm), C65 carbon, and PVdF binder. The cycled electrode shows a different top-down surface morphology (Figure 5b). In this case, a dense and rather smooth surface film is formed. The FIB-polished cross section of the pristine electrode (Figure 5c) reveals the interconnected electrode porosity. Figure 5d shows the details of the SEI formed on the electrode’s surface in the cycled cell. This surface SEI is dense and smooth. Moreover, it is also formed inside the bulk of the electrode, filling the interconnected electrode porosity.^49^ Consequently, the growth of the SEI inside the electrode inevitably leads to the blocking of ionic pathways throughout the cycling process. This led to worse electrochemical performance, as observed in Figure 4d. It has to be noted that the particle’s size distribution within the bulk electrode, as presented in Figure 5d, is not illustrative. This is due to the random choice of the FIB cross-section positioning on the electrode that has a large and nonhomogeneous particle size distribution (Figure S11). From the particle size distribution of Corn@1400 °C (Figure S11), it is evident that most particles are of the size of around 100 nm but with a large scatter in distribution with some particles of the size of 10 μm.

**Figure 6:**
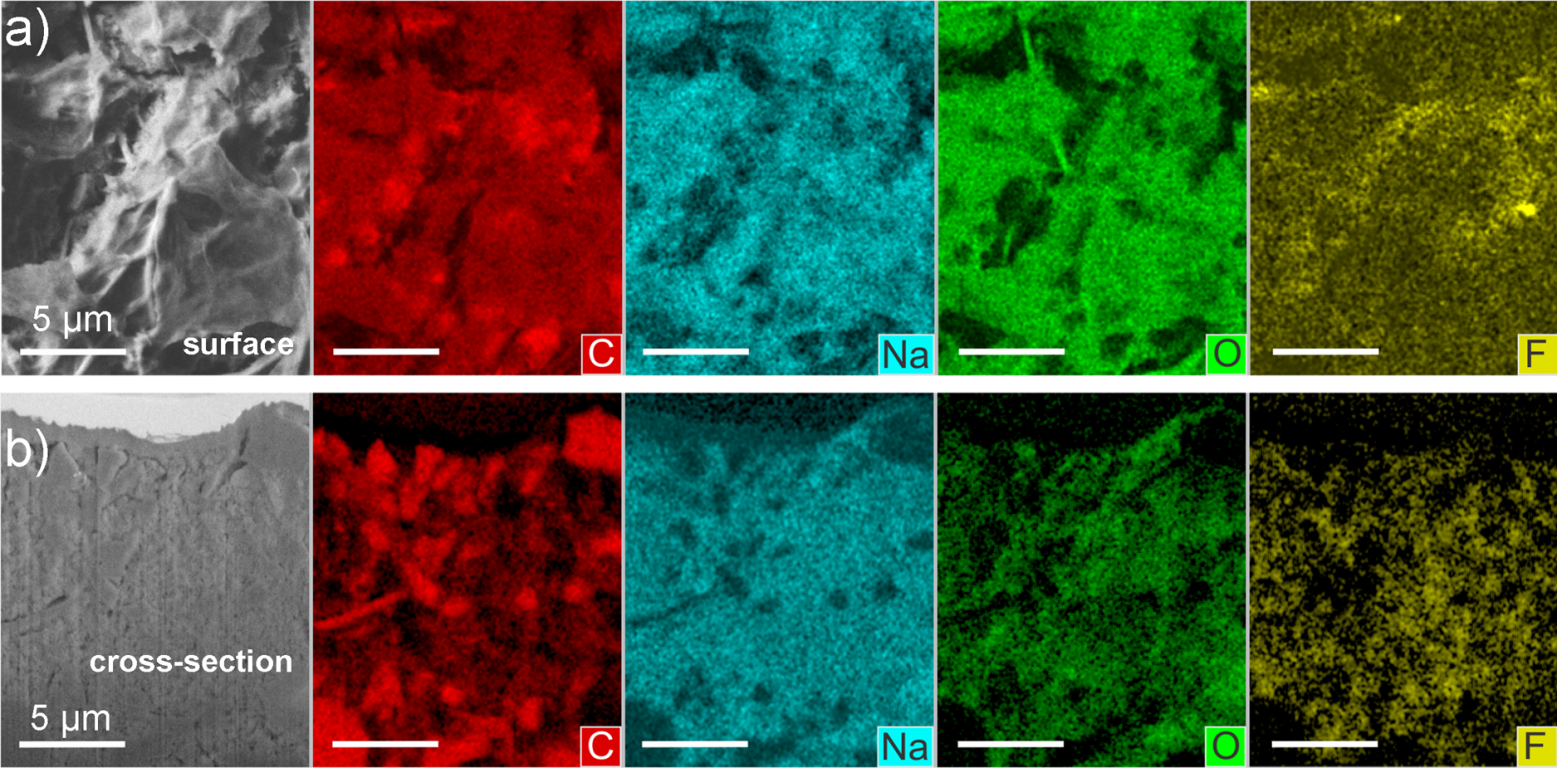
a) Surface SEM analysis with the corresponding EDX elemental distribution maps and (b) FIB-polished cross section with the corresponding EDX elemental distribution maps of sample.

The elemental distribution and composition of the SEI was probed by SEM–EDX. The elemental composition of the pristine electrode (Figure S12) is discussed in Note 2 in the Supporting Information. Figure 6 shows the EDX elemental distribution maps of surface SEM as well as the FIB-polished cross section. The EDX mapping for the cycled cell confirms the presence of SEI both at the surface and inside the bulk of the electrode via the presence of Na intensity (Figure 6a,b). Although the surface SEI in Figure 6a shows lower Na and O intensity compared to the bulk electrode SEI, the quantitative EDX spectra from the selected areas (Figure S13) reveal that the surface SEIs have 41.8 ± 3.5 wt % Na and 19.4 ± 1.8 wt % O while the bulk electrode SEIs have 33.2 ± 0.5 wt % Na and 16.5 ± 0.4 wt % O. Such a discrepancy between the mapping intensities and quantification results is due to the small thickness of the surface SEI, which results in a smaller analytical volume of the containing elements. The F amount is due to its presence in the PVdF binder used for the electrode.

**Figure 7:**
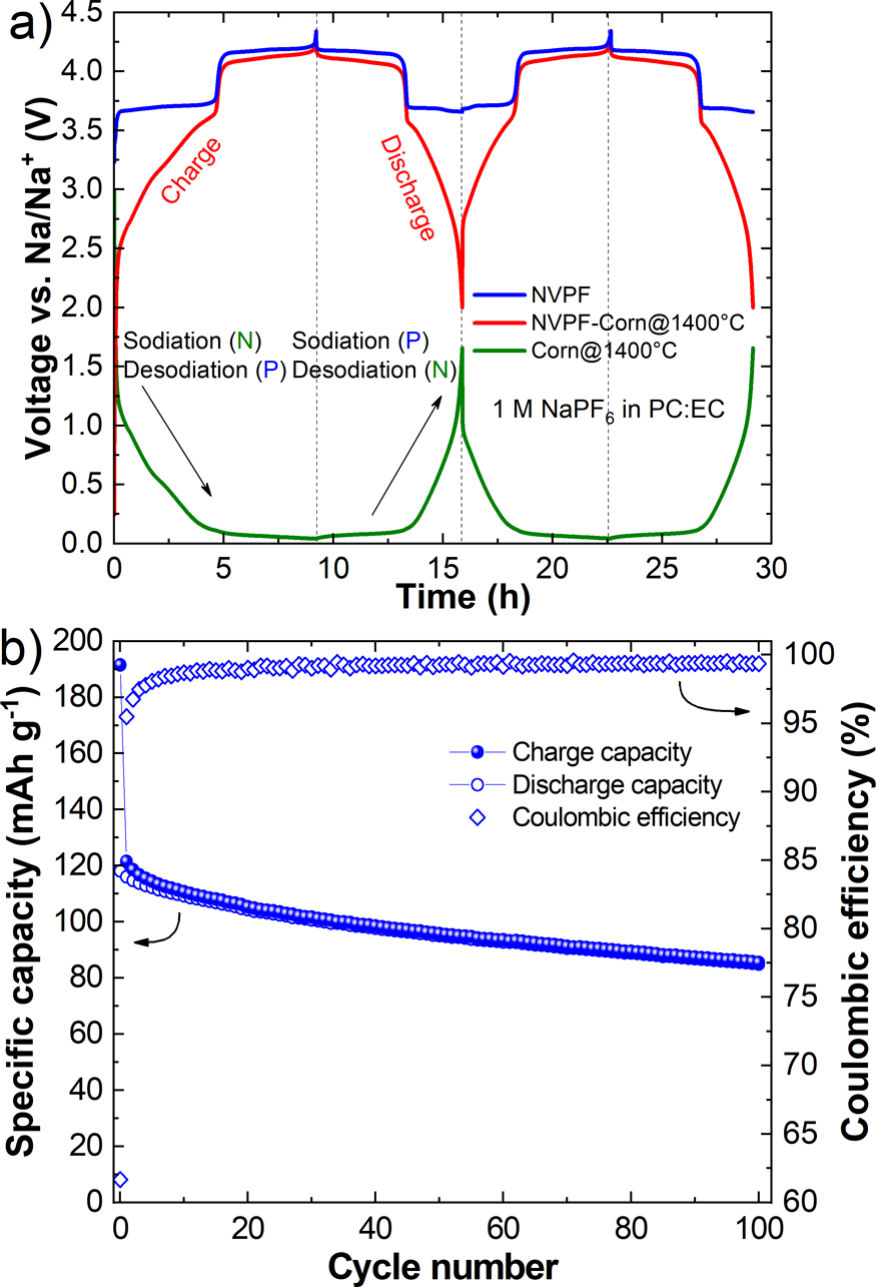
a) Electrochemical performance of formation cycles of the NVPF/Corn@1400 °C three-electrode full cell and (b) cycling stability over 100 cycles of the NVPF/Corn@1400 °C two-electrode cell.

To study the adequacy of the prepared corncob-derived non-graphitizable carbons, three-electrode full cell measurements were performed. This approach is essential for clear separation of the electrochemical contribution of the negative and positive electrodes. This helps to determine the limiting electrode in the battery. Based on the results of the half-cell measurements (Figure 4d), Corn@1400 °C was selected as the negative electrode. Commercial NVPF [Na_3_V_2_(PO_4_)_2_F_3_] was used as the positive electrode and Na metal as the reference electrode. Figure 7a presents the electrochemical behavior of the first two cycles. In the first desodiation step (relative to the positive electrode), two characteristic plateaus are observed, corresponding to the extraction of two Na^+^ ions per formula unit from the NVPF. Simultaneously, the sodiation of the negative electrode takes place. In the first sodiation step (relative to the negative electrode), both of the characteristic features of Na^+^ insertion into the non-graphitizable carbons, namely, the sloping and the plateau region, can be clearly distinguished. Additionally, a knee is observed at around 500 mV, corresponding to the electrolyte decomposition on the carbon surface. Meanwhile, in the reinsertion step of the positive electrode, it can be seen that only one of the plateaus at a higher voltage is completed, while the second plateau is only partially finished. This means that the reinsertion of Na^+^ ions back into the positive electrode (NVPF) is not complete, resulting in an irreversible loss of the active material. Considering the shorter length of the desodiation plateau for the negative electrode compared to that of sodiation (Figure 7a), it can be assumed that not all of the inserted Na^+^ could have been extracted from the negative electrode upon the desodiation step. A possible explanation is the loss of Na^+^ to the formation of the SEI. Another possibility could be the trapping of Na^+^ in the pores of the material.^50^ A similar behavior was observed in half-cell experiments (Figure 4). Finally, no additional loss of Na^+^ is observed in the second cycle.

Cycling stability of a two-electrode full cell over 100 cycles is presented in Figure 7b. The cell exhibits a high charge capacity of 191 mAh g^–1^ while also displaying high irreversible capacity in the first cycle. Consequently, the iCE amounts to 61%. Afterward, the capacity slowly decreases, reaching 85 mAh g^–1^ after 100 cycles. The Coulombic efficiency is low in the initial cycles, reaching 99% only after 30 cycles (Figure S14a) and then stabilizing between 99.3 and 99.5%. Electrochemical curves representing the selected cycles are presented in Figure S14b. In the first cycle, both of the plateaus corresponding to the desodiation/sodiation step of the NVPF electrode are clearly visible. In subsequent cycles, these two plateaus get less pronounced while also contributing less capacity.

## Conclusions

Corncob-derived non-graphitizable carbons were prepared at different carbonization temperatures by a two-step process to establish correlations between structural properties and electrochemical behavior. The corncob constituents were determined to be a mixture of cellulose and hemicellulose. A detailed investigation of structural properties was carried out on different size scales with complementary techniques, SWAXS, STEM, and EELS, providing structural information on a nanometer scale. Several unconventional parameters such as the correlation length beyond which the order is lost, the fractal cut-off length, and the lengths of locally flat regions insinuated the trend of increased ordering of the carbon matrix with the increased temperature of carbonization. The obtained SWAXS parameters were more explicitly shown in TEM images, where the increased stacking and the decreased curvature of graphene layers could be observed. Ordering of the structure and increase of conductivity were additionally confirmed by the increasing *sp^2^*/*sp^3^* ratio determined by EELS.

The presence of ultramicroporosity was determined by SWAXS, while N_2_ gas adsorption provided complementary data on a micro-/mesoscale. The increase of the specific surface area of micropores determined from SWAXS data is in correlation with the iCEs, a larger surface area leading to lower efficiencies. The capacity contributions show a clear dependence between the structural properties of carbons and the ratio between the sloping and plateau regions. Furthermore, parameters determined by SWAXS imply that the contribution of the plateau region increases with the increasing temperature of carbonization. Meanwhile, Raman spectroscopy, EELS, and the parameter *w*_L_ from SWAXS indicate the presence of defects in the carbon matrix, suggesting a higher contribution of the sloping region in the galvanostatic curves at lower temperatures of carbonization.

Based on the performance of prepared carbons in the half-cell experiments, Corn@1400 °C was selected as the negative electrode in the three-electrode full cell configuration. In the first sodiation step of the carbon electrode, we were able to clearly distinguish the sloping and the plateau region, and the desodiation of the positive electrode was completed. In the desodiation step of the carbon electrode, we observed the plateau region attributing to a lower capacity than in the sodiation step, indicating diffusional limitations. Consequently, the reinsertion of sodium into the positive electrode was not completed. These diffusional limitations were elucidated with FIB-SEM analysis of electrodes cycled in half-cells, where it was identified that the SEI grows on the surface as well as inside the bulk of the electrode, blocking the ionic pathways.

In summary, combined N_2_ adsorption, SWAXS, TEM, FIB-SEM, and electrochemical studies of corncob-derived nongraphitizable carbons exposed the complex interdependencies of structural, morphological, and electrochemical properties on the battery performance.

## Methods

### Materials

Non-graphitizable carbons were obtained by two-step pyrolysis of corncob and obtained from a local farmer. Grains of corn were removed from the cob, which was later broken into smaller pieces and dried in a vacuum dryer at 80 °C overnight. First, heat treatment was carried out in a quartz tube furnace (under argon flow, 15 L h^–1^) up to a temperature of 900 °C with a heating rate of 2.5 °C min^–1^. The temperature was held at 900 °C for 1h and then cooled to room temperature. A second step was performed up to 1200, 1400, and 1600 °C in an Al_2_O_3_ tube furnace (under argon flow, 100 L h^–1^) with a heating rate of 2.5 °C min^–1^ and holding at constant temperature for 1 h. The heat treatment process flow rate is shown in Figure S15. The obtained non-graphitizable carbon samples were denoted as Corn@900, Corn@1200, Corn@1400, and Corn@1600.

### Characterization

Thermogravimetric (TG) measurements were performed on a Netzsch 449 F3 Jupiter instrument under a dynamic Ar (5.0) flow with a flow rate of 50 mL min^–1^ in a temperature range from 30 °C to 1200 °C. A heating rate of 10 K/min was used. About 10 mg of sample was placed in an alumina (Al_2_O_3_) crucible. Simultaneously, mass spectrometry was performed on a MS 403C Aëolos with a SEM Chenneltron detector and a system pressure of 2 × 10^–5^ mbar. Gasses that evolved under TG heat treatment were transferred to the mass spectrometer through a transfer capillary, quartz ID: 75 μm, which was heated up to 220 °C. The upper limit of the mass spectrometer detector was 100 AMU.

The crystal structure of the samples was characterized by X-ray powder diffraction (XRD). Measurements were carried out on a PANalytical X’pert PRO high-resolution diffractometer with Cu Kα_1_ radiation (λ = 1.5406 Å) in the range of 2θ from 10 to 80° with a step of 0.033° and a measurement time of 1 s per step. Values of 2θ were converted to the values of the scattering vector, *q,* for the unification of scale between XRD and SAXS measurements. Converted values of *q* range from 7.1 to 52.4 nm^–1^.

Raman spectroscopy measurements were carried out using a wavelength of 532 nm (WITec Alpha300 SRA Plus, WITec GmbH). The laser intensity was 1.5 mW to avoid any laser-induced changes. Each of the samples was measured on four different places. More details are provided in Note 1 in the Supporting Information.

Small-angle X-ray scattering (SAXS) measurements were performed on an in-lab-modified old Kratky-type camera (Anton Paar) on an X-ray generator (Seifert, ID3003) with the Cu-anode X-ray tube (Cu *K*_α_ line) operating at 40 kV and 50 mA. We have provided a detailed description of the experimental and theoretical procedures and methods in the Supporting Information.^39,40,51−56^

The N_2_ gas adsorption analysis was done using a Micromeritics ASAP 2020 apparatus. Surface area analysis was done using the Brunauer–Emmett–Teller (BET) method, while the Barrett–Joyner–Halenda (BJH) method was used to evaluate the pore size distribution.

SEM (FE-SEM, Supra 35 VP Carl Zeiss) was used for the characterization of the morphology of the samples.

The materials morphology and particle’ distribution were examined using a JEM-ARM200CF, probe C_s_-corrected scanning transmission electron microscope, equipped with a cold field emission gun (FEG) electron source operated at 80 kV, a JEOL Centurio 100 mm^2^ EDXS detector and a JEOL STEM detector (JEOL, Tokyo, Japan), and a GIFQuantum ER dual-EELS system (GATAN-AMETEK, Pleasanton, USA), which was used for electron energy loss spectroscopy (EELS). TEM samples were prepared by dispersing approximately 300 μg of the material in 5 mL of absolute ethanol in an ultrasonic bath for 15 min. Then, 20 μL of the suspension was placed on the copper TEM grid. For materials imaging, high-resolution STEM high-angle annular dark-field/bright-field (STEM-HAADF/-BF) modes were used. The EELS measurements of C K edge were performed at 80 keV accelerating voltage with 0.25 eV energy dispersion in a spectrum imaging mode to reduce the possible material damage. Spectra were then summed over the whole spectrum imaging area and deconvolved to remove plural scattering. The height and the area of the π* peak in EELS C K edge of graphite-like carbon nanomaterials are dependent on the orientation of the graphene layers toward the electron beam, that is, whether the beam propagates through the sample along the (002) plane or is perpendicular to it.^57^ To overcome this directionality effect and make spectra more directly comparable, the EELS spectral imaging was done from the larger analysis areas of about 30 × 30 nm. The π*/(π*+σ*) ratio used for assessing the *sp^2^* content of the samples was calculated from C K edge spectra using an energy window of 1 eV centered at 285 eV π* peak for π* and at 20 eV starting at 284.5 eV for (π* + σ*).^43^

Cross-sectional analysis was carried out in a focused ion beam-scanning electron microscope Helios Nanolab 650 (FEI, Netherlands) equipped with a vacuum transfer interlock (Gatan, US) and an energy-dispersive X-ray (EDX) spectrometer X-MAX 50 (Oxford, UK). Samples were mounted on an Al shuttle stub and assembled into an ALTO 1000 transfer holder (Gatan, US) inside an Ar-filled glovebox and transferred under vacuum conditions directly into an FIB-SEM chamber. Detailed morphological images with phase contrast information were obtained by in-column SE/BSE detectors at low-energy pre-monochromated electron beam (1 kV @ 50 pA, UHR, U-mode). EDX quantitative analysis and elemental distribution maps were acquired at 7 kV with the beam current set to 0.8 nA. A more detailed explanation of the preparation methods is described in Note 2 of Supporting Information.

### Electrode Preparation

Prior to electrode preparation, the samples were ground in a mixer mill (SPEX SamplePrep, Retsch) for 30 min. Electrodes were prepared with a composition of 90 wt % non-graphitizable carbon material, 5 wt % conductive carbon Super C65 (Timcal), and 5 wt % polyvinylidene fluoride (PVdF, Aldrich). The mixture was dissolved in *N*-methyl pyrrolidone (NMP, Aldrich) and ball milled for 30 min at 300 rpm to obtain a homogeneous slurry. The slurry was then cast on a carbon-coated Al foil (Armor, France) with a doctor blade applicator with a thickness of 100 μm. The coated slurry was dried at 80 °C overnight. Electrodes with a diameter of 16 mm were punched out the next day and transferred to an argon-filled glovebox. The loadings were maintained between 1.5 and 2 mg cm^–2^.

### Cell Assembly and Electrochemical Measurements

The electrochemical measurements were conducted in two-electrode pouch-type cells. The cells were assembled in an argon-filled glovebox with water and oxygen contents below 0.5 ppm. Non-graphitizable carbon electrodes were used as working electrodes, whereas sodium metal (Aldrich, approximately 500 μm thick) was used as the counter electrode. Both electrodes were separated by a glass fiber separator (Whatman, GF-A). The electrolyte used was 1 M NaPF_6_ in a solvent mixture of propylene carbonate (PC) and ethylene carbonate (EC) (1:1 vol. %). Each cell was filled with 80 μl of electrolyte. Electrochemical measurements were carried out within a potential window between 0.005 and 2.5 V versus Na/Na^+^ employing the following protocol: five formation cycles with a current of 30 mA g^–1^ (theoretical capacity was taken according to the model proposed by Bommier,^13^ i.e., 301.6 mA h g^–1^), which roughly translates into a rate of C/10. After the initial 5 cycles, the current was changed to a higher rate of 300 mA g^–1^ (corresponding to a rate of 1C) and measured for another 100 cycles. At the end of each discharge, a constant voltage step was applied at the lower cut-off, limited to 15 h, or until the current rate was lower than C/100. For the Na metal deposition measurements, the cells were cycled within a potential window between −0.2 and 2 V versus Na/Na^+^. Two cells were assembled to perform measurements at different currents at C/10 and at 1 C. Only the first cycle was performed in both cases. When the potential reached negative values, a time-limiting step was introduced, concluding the sodiation step after 15 h.

Three-electrode cells (Hohsen Corp., Japan) were assembled in a full cell configuration. The positive electrode (WE) was Na_3_V_2_(PO_4_)_2_F_3_ (NVPF) obtained from TIAMAT, France. The negative electrode (CE) was Corn@1400 °C and Na metal was used as the reference electrode (RE). The areal capacity ratio between the negative and positive electrodes was maintained at 1.2:1. Both electrodes were separated by a glass fiber separator (Whatman, GF-A) wetted with 130 μl of 1 M NaPF_6_ in the mixture of propylene carbonate (PC) and ethylene carbonate (EC) (1:1 vol %). Electrochemical measurements were carried out within potential windows of 2.0 and 4.35 V versus RE. Only the measurements of formation cycles were performed to obtain information about the sodiation/desodiation process taking place at the non-graphitizable carbon electrode. Additionally, two-electrode cells were assembled in a full cell configuration to obtain information on cycling stability over 100 cycles. NVPF was used as the positive electrode (WE) and Corn@1400 °C was used as the negative electrode (CE). Both electrodes were separated by a glass fiber separator (Whatman, GF-A) wetted with 100 μL of 1 M NaPF_6_ in the mixture of propylene carbonate (PC) and ethylene carbonate (EC) (1:1 vol %). Electrochemical measurements were carried out within potential windows of 2.0 and 4.35 V. The cell was cycled at the rate of C/10.

All the half-cell electrochemical measurements were performed using a Maccor Series 4200 potentiostat/galvanostat (Maccor, Inc). The full cell electrochemical measurements were performed using a Biologic VMP3. All experiments were conducted at room temperature of 25 °C.

## Supporting Information Available

Experimental and methods, CHNS elemental analysis, XRD patterns, adsorption isotherms, SWAXS curves, structural parameters, Raman spectra, SEM images, intensity line profiles, *sp^2^*/*sp^3^* ratios, iCE, voltage profile galvanostatic curves, statistical analysis, FIB-SEM images, EDX spectra, and heat treatment process flow rate (PDF)
