# Supplementary material for: Correlating Structural Properties with Electrochemical Behavior of Non-graphitizable Carbons in Na-Ion Batteries
Source: ACS Appl Energy Mater. 2022 Aug 23;5(9):10667–79. doi: 10.1021/acsaem.2c01390 (PMC9516555; doi:10.1021/acsaem.2c01390)
Supplement: Supplementary file 1 — ae2c01390_si_001.pdf [file ae2c01390_si_001.pdf]

SUPPORTING INFORMATION:

Correlating structural properties with electrochemical behavior of non-graphitizable carbons in Na-ion batteries

Blaž Tratnik,[a,b] Nigel Van de Velde,[a] Ivan Jerman,[a] Gregor Kapun,[a] Elena Tchernychova,[a] Matija Tomšič,[b] Andrej Jamnik,[b] Boštjan Genorio,[b] Alen Vizintin,[a,*] Robert Dominko.[a,b,c]

[a] *National Institute of Chemistry, Hajdrihova 19, 1000, Ljubljana, Slovenia*
[b] *Faculty of Chemistry and Chemical Technology, University of Ljubljana, Večna pot 113, 1000, Ljubljana, Slovenia*
[c] *ALISTORE-European Research Institute, CNRS FR 3104, Hub de l'Energie, Rue Baudelocque, 80039, Amiens Cedex, France*

Corresponding Author: alen.vizintin@ki.si

Experimental and Methods

1.1 X-ray powder diffraction measurements

Raw data from XRD measurements was fitted with Origin software and all of the calculated structural parameters were based on the results of the fitting. Raw data and its corresponding fits are shown in Figure S2.

The interlayer spacing was calculated from Bragg's law:[1]

$$d = \frac{2\pi}{q} \quad \text{(eq. S1)}$$

The height of graphene layer stack, L_C is calculated by:[2]

$$L_C = \frac{0.9 * \lambda}{FWHM(002) * \cos\theta(002)} \quad \text{(eq. S2)}$$

where λ is the wavelength and FWHM is the full width half maximum value corresponding to the (002) basal plane.

Average number of graphene layers is determined as the ratio of the height of the graphene layer stack and the interlayer spacing $(L_C/d) + 1$.

1.2 Raman spectroscopy – defined the concentration of defects

To quantify the structural order with Raman spectroscopy, we defined the concentration of defects (α) as:[3]

$$\alpha = \frac{I_D}{I_G} \quad \text{(eq. S3)}$$

where I_G is the integrated area of the G-band (~1580 cm^{-1}) and I_D is the area of the D band (~1350 cm^{-1}).

1.3 Small-Angle X-Ray Scattering Measurements

The SAXS measurements were performed by an in-lab-modified Kratky-type camera (Anton Paar KG, Graz, Austria), which was attached to the conventional generator (GE Inspection Technologies, SEIFERT ISO-DEBYEFLEX 3003) with a Cu-anode operating at 40 kV and 50 mA (Cu-K_α line with λ = 1.54 Å). The camera was equipped with a Goebel mirror (Osmic MAX-FLUX# focusing multilayer optics), which served as a monocromator and focusing element for the primary X-ray beam, and a block-collimation unit, which provided a well-defined, line-collimated primary beam. Correspondingly, the obtained SAXS data were experimentally smeared. The samples were placed between the two sheets of Scotch® tape in

a 0.5 mm thick sample holder and were positioned in the primary beam in an evacuated SAXS camera (pressure between 2 and 4 mbar). The measurements were performed at 25°C with the total sampling time of 3 minutes per sample utilizing the Mythen 1K microstrip solid-state diode-array detector (Dectris, Baden, Switzerland) in the small-angle regime of scattering vectors from $0.08 < q < 7$ nm^{-1}, where $q = (4\pi/\lambda)\cdot \sin(\vartheta/2)$. The SAXS data were subsequently corrected for the Scotch® tape and background scattering and were desmeared utilizing the so-called primary beam width and length profiles and the well-known iterative desmearing procedure introduced by Lake.[4] The desmeared SAXS data was then put to the absolute scale using water as a secondary standard[5] and further extended by the XRD data to obtain the overall SWAXS curves in the regime of the scattering vectors from $0.08 < q < 24$ nm^{-1}. These SWAXS curves were on absolute scale and were in units of cm^{-1}.

1.4 Small- and Wide-Angle X-Ray Scattering Data Fitting

The SWAXS data of the non-graphitizable carbon samples studied were fitted according to the approaches described by Saurel et al. in ref. [6]. For the convenience of the interested reader, we provide below only a brief explanation of the fitting procedure used and encourage the reader to search the original publication for the details. In the framework of this procedure, the total SWAXS scattering intensity can be described as the sum of three different scattering contributions covering the three different characteristic length scales that can be resolved from the hierarchical structure of the studied non-graphitizable carbon samples (Figure 2 in main article). Region I of this hierarchical structure is micrometer-sized carbon particles with a more or less rough surface. Region II is the nanometer-sized pores inside these microparticles, and the most detailed region III is the lamellar or so-called crumpled lamellar structure of carbon on the atomic scale. The total SWAXS scattering intensity, I_{SWAXS}, in units of cm^2 g^{-1} can be written as:[6]

$$I_{\mathrm{SWAXS}} = I_{\mathrm{Porod}} + I_{\mathrm{mp}} + I_{\mathrm{WAXS}}, \qquad \text{(eq. S4)}$$

where I_{Porod} denotes the scattering contribution representing the Porod scattering regime of the micrometer-sized carbon particles (powder grains) with well-defined interphase surface, I_{mp} the scattering contribution of the micropores, their clusters and arrangement inside the carbon particles, and I_{WAXS} the scattering contribution of the lamellar arrangements of the carbon atoms.

The first contribution in eq. (S4) can be written as:[6]

$$I_{\text{Porod}} = 2\pi\left(\Delta SLD_{\text{S}}\right)^2 \left(S_{\text{macro}}\, q^{-4} + S_{\text{rough}} \frac{\frac{2}{9} R_{\text{rough}}^4}{1 + \frac{1}{5}\left(qR_{\text{rough}}\right)^2 + \frac{2}{9}\left(qR_{\text{rough}}\right)^4} \right), \qquad \text{(eq. S5)}$$

where ΔSLD_{S} represents the scattering length density of the powder grains (considering the volume fraction of the pores inside) in respect to the surrounding vacuum, which is related to the scattering contrast of these carbon powder grains, S_{macro} the macroscopic specific surface area of the powder grains, S_{rough} the contribution to the specific surface area due to the fine roughness of the grain surface, R_{rough} the characteristic size of the grain surface roughness, and q the length of the scattering vector.

The second contribution in Eq. (S4) can be written in a form of the well-known semi-empirical Teubner-Strey model,[7,8] which was initially derived for the microemulsion systems and can be written as the following set of equations:[6]

$$\begin{aligned} I_{\text{mp}} &= I_{\text{o}} \cdot \frac{1}{1 + C_1\, q^2 + C_2\, q^4} \\ I_{\text{o}} &= \frac{8\pi}{\rho_{\text{struc}}} \cdot \phi \cdot \left(\Delta SLD\right)^2 \cdot \frac{\xi^3}{\left(1 + \left(2\pi\xi/d\right)^2\right)^2} \\ d &= 2\pi \left(\frac{1}{2} C_2^{-\frac{1}{2}} - \frac{C_1}{4C_2} \right)^{-\frac{1}{2}} \\ \xi &= \left(\frac{1}{2} C_2^{-\frac{1}{2}} + \frac{C_1}{4C_2} \right)^{-\frac{1}{2}} \end{aligned}, \qquad \text{(eq. S6)}$$

where ρ_{struc} is the structural density of the non-graphitizable carbon samples, ϕ_{pores} is the volume fraction of nanometer-sized micropores, ΔSLD is the scattering length density of carbon with respect to vacuum, which is related to the scattering contrast of the pores and is related to ΔSLD through the relation $\Delta SLD_{\text{S}} = \Delta SLD \cdot (1-\phi)$; d is the pore-to-pore distance, and ξ is the characteristic correlation length of the spatial pore distribution. This model was derived for the two-phase system with the characteristic mean distance between the domains of the two phases, d, where the parameter f_{a} represents the so-called amphiphilic factor, which in this case can be interpreted as related to disorder in the system:[6]

$$f_{\text{a}} = \frac{C_1}{2\sqrt{C_2}} \qquad \text{(eq. S7)}$$

Based on this scattering contribution one can also estimate the radius of the micropores, r, and the surface area of the pores, S_{mp}:[6]

$$r=\sqrt{5\cdot C_1}$$
$$S_{\mathrm{mp}}=\frac{I_{\mathrm{o}}}{2\pi\, C_2\,(\Delta SLD)^2} \qquad \text{(eq. S8)}$$

At this point we must stress that the average pore radius calculated in this way is accurate only for spheroid pores in the low pore concentration limit, when f_{a} = 0.4 (i.e. when $C_2\approx 2.8\cdot C_1$). More generally, on the basis of the Babinet principle[9] an expression for the average pore-width, w_{p}, and an estimate of the average width of the carbon matrix, w_{C}, can be obtained from the Teubner-Stray form factor according to the expressions:

$$w_{\mathrm{p}}=\frac{\xi}{1-\phi}$$
$$w_{\mathrm{C}}=\frac{\xi}{\phi} \qquad \text{(eq. S9)}$$

Parameters w_{p} and w_{C} are the pore and carbon matrix effective lengths, respectively, which can be considered as a good estimation of their respective average widths.

The third contribution in eq. S4 can be written as:[6]

$$I_{\mathrm{WAXS}}=K\cdot\frac{S_{\mathrm{3D}}}{q^2}\cdot L(q_{\mathrm{c}},w_{\mathrm{L}})\otimes G(q_{\mathrm{c}},w_{\mathrm{G}})\cdot e^{-q^2\langle\delta z^2\rangle/3}$$
$$\frac{S_{\mathrm{3D}}}{q^2}=\left(1+\frac{D}{r^D}\cdot\Sigma^D\cdot\frac{D\,\Gamma(D-1)}{\left(1+(q\,\Sigma)^2\right)^{\frac{D-1}{2}}}\cdot\frac{\sin\left((D-1)\cdot\tan^{-1}(q\,\Sigma)\right)}{q\,\Sigma}\right)\cdot$$
$$\cdot\left(e^{-(qr)^2/6}+\left[erf\left(\frac{1.06}{2\sqrt{3}}\cdot qR\right)\right]^6\cdot\frac{4}{(qR)^2}\right), \qquad \text{(eq. S10)}$$

where K is an intensity scaling factor, $L(q_{\mathrm{c}},w_{\mathrm{L}})\otimes G(q_{\mathrm{c}},w_{\mathrm{G}})$ the Voigt peak profile function, $\langle\delta z^2\rangle$ the local fluctuation of the interlayer distance due to the local distortions of the structure,[10] R the length below which the layers can be considered as effectively flat, Σ the fractal cut-off length – above the curvature becomes random; and D the fractal dimension. The Voigt peak profile function accounts for the long-range distortions in the crystallite structure[10] and is a convolution of Lorentzian, L, and Gaussian, G, functions, with w_{L} and w_{G} the

respective full widths at half maximum and q_c the position of the peak center, corresponding to $q_c = 2\pi/d$, d being the structural periodicity.

In order to use Eq. (S4-S6) and (S10) to fit the experimental SWAXS data, it was first necessary to determine the values of the parameter ΔSLD and its linear relationship with the density of non-graphitizable carbons. Based on the XRD data, which provided the two characteristic XRD peak position values, q_{002} and q_{100}, for the reference graphite (ρ_{graphite} = 2.255 g cm^{-3}) and the non-graphitizable carbon samples, the structural density of the non- graphitizable carbons, ρ_{struct}, was calculated according to the relationship:[6,11]

$$\rho_{\text{struct}} = \rho_{\text{graphite}} \cdot \frac{d_{002,graphite}}{d_{002}} \cdot \left(\frac{d_{100,graphite}}{d_{100}}\right)^2 , \qquad \text{(eq. S11)}$$

where $d_i = 2\pi/q_i$. The values of ρ_{struct} and the NIST online tool[12] were used to calculate the corresponding values of ΔSLD. The parameters ϕ_{pores} and ΔSLD_S were determined simultaneously by the data fitting procedure. The sample density, ρ_{sample}, of the studied non-graphitizable carbons was determined according to the following relation:[6]

$$\rho_{\text{sample}} = \rho_{\text{struct}} \cdot (1-\phi) \qquad \text{(eq. S12)}$$

The values of these structural parameters are gathered in Table S2.

Our experimental SWAXS curves were given in units of cm^{-1} and would have to be normalized by the sample density to obtain the total SWAXS scattering intensity, I_{SWAXS}, curves in units of cm^2 g^{-1}. The sample density of the non-graphitizable carbon sample is difficult to determine because it depends on the volume fraction of the pores, which is a fitting parameter. However, according to Eq. (S11), the determination of the structural density is quite straightforward. Therefore, in our study we fitted the SWAXS curves in units of cm^{-1} and multiplied the right-hand side of Eq. (S4) with the Eq. (S12) accordingly.

Table S1: CHNS Elemental analysis of corncob derived non-graphitizable carbons prepared at different temperatures of carbonization.. The sum of weight fractions does not add up to 100% because anorganic impurities and O could not be determined with this technique

Sample	C [wt. %]	H [wt. %]	N [wt. %]	S [wt. %]
Corn@900°C	87.9	0.9	1.2	<0.01
Corn@1200°C	93.1	0.2	0.8	<0.01
Corn@1400°C	96.4	0.1	0.3	<0.01
Corn@1600°C	96.9	0.1	0.2	<0.01

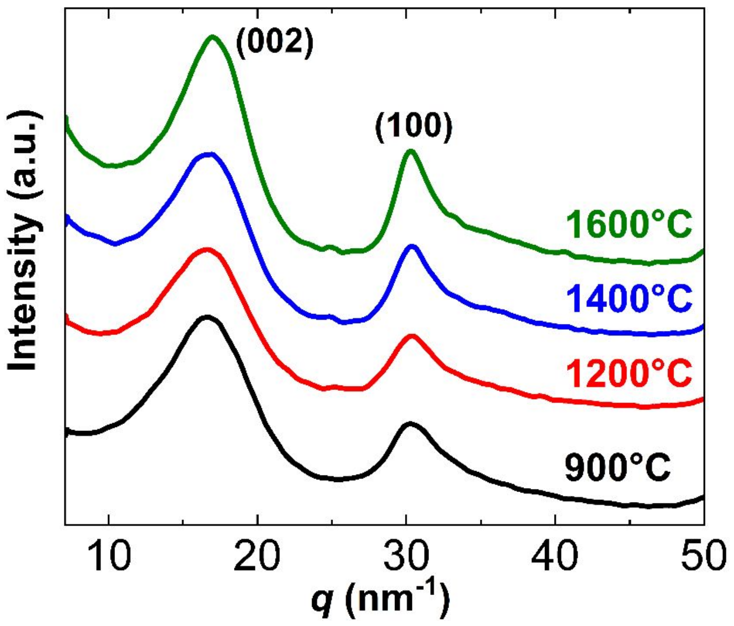

Figure S1: XRD patterns of corncob derived non-graphitizable carbons prepared at different temperatuers of carbonization. The (002) and (100) characteristic peaks are designated on the graph.

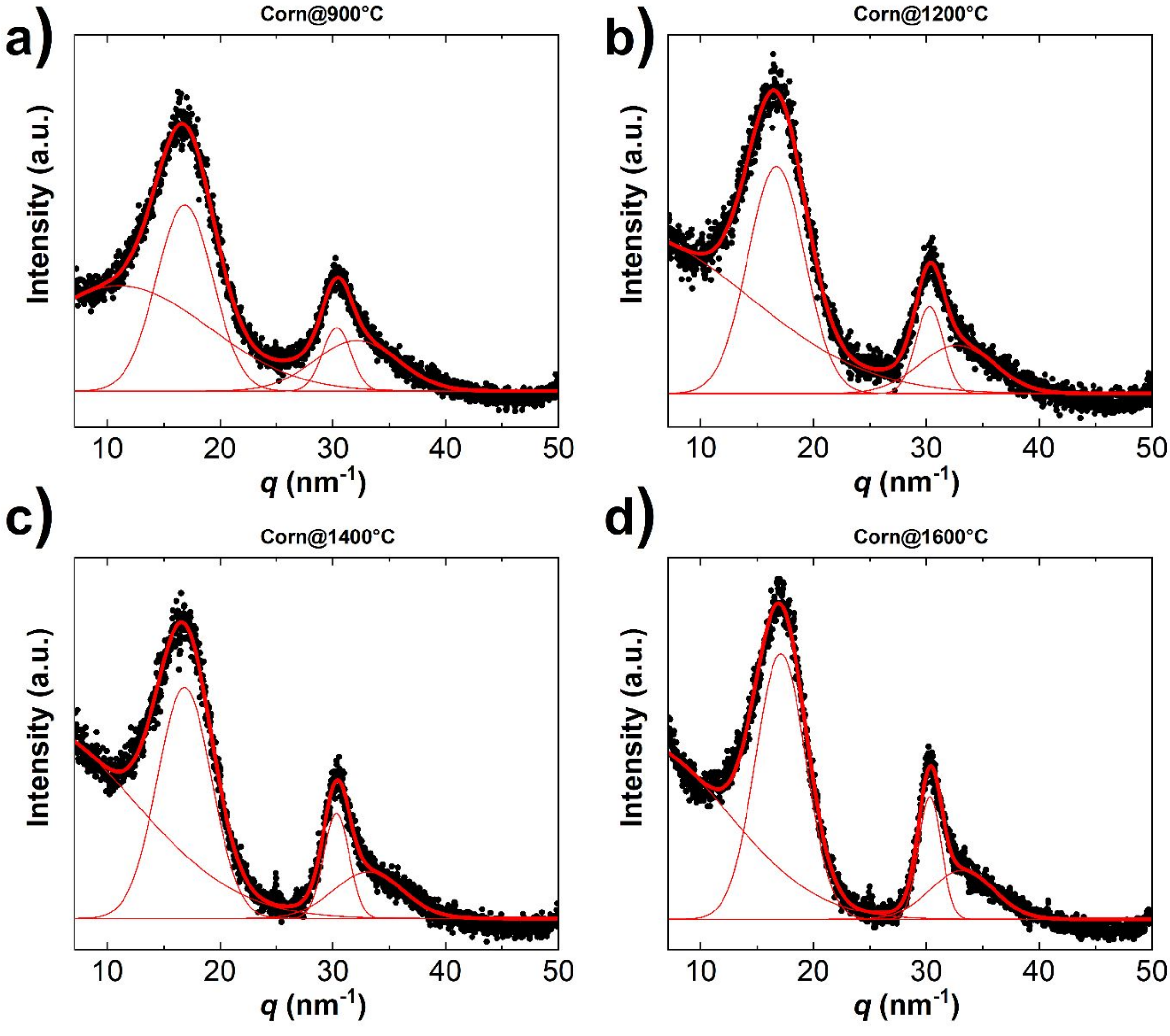

Figure S2: XRD measurements and the corresponding fits for a) Corn@900°C, b) Corn@1200°C, c) Corn@1400°C, d) Corn@1600°C

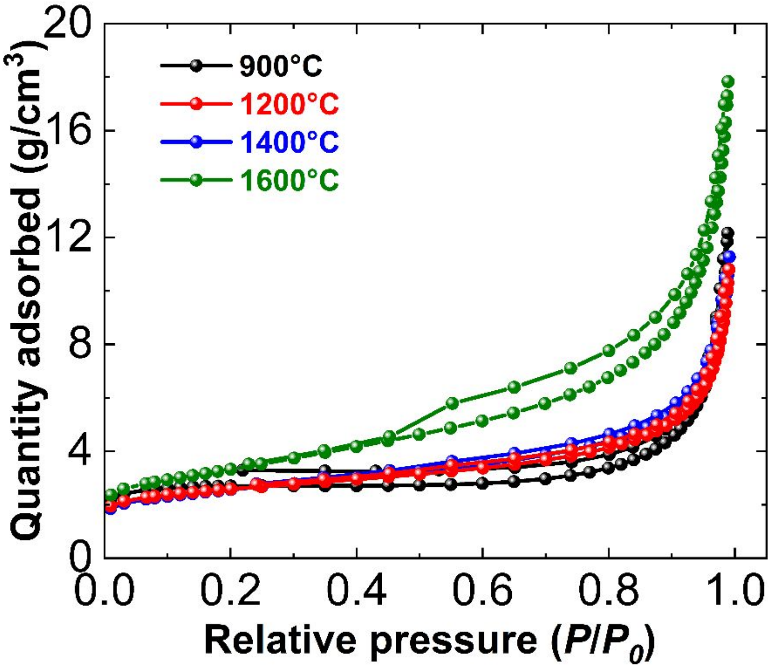

Figure S3: Adsorption isotherms of corncob derived non-graphitizable carbons. The adsorption measurements were performed with N_2 as an adsorptive gas.

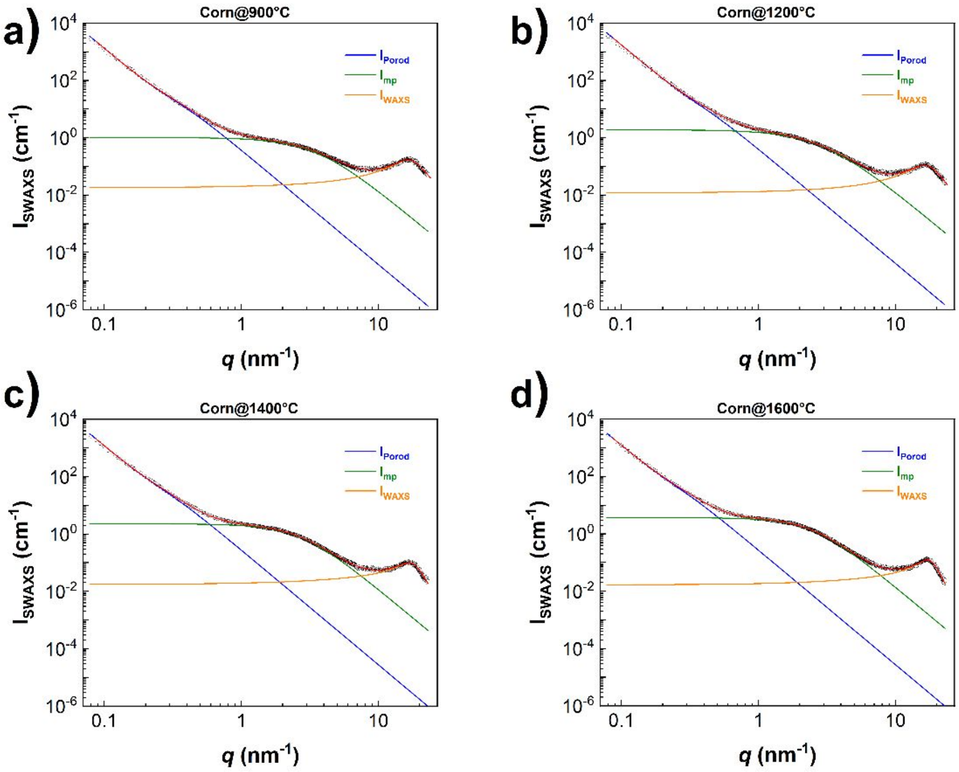

Figure S4: Experimental small- and wide-angle X-ray scattering curves of (a) Corn@900°C, (b) Corn@1200°C, (c) Corn@1400°C and (d) Corn@1600°C. Fits of individual scattering contributions are also presented: I_{Porod} *(blue) denotes the scattering contribution of micrometer-sized particles with well-defined interfaces to either other particles or vacuum,* I_{mp} *(green) denotes the contribution from micropores, their clusters and arrangements inside the carbon particles,* I_{WAXS} *(orange) denotes the lamellar arrangements of the carbon atoms, and the total fitting curve (red) representing the sum of these three scattering contributions.*

Table S2: Structural parameters deduced from the XRD results obtained for the reference graphite and the studied non-graphitizable carbon samples, where q_{002} is the position of the (002) peak, q_{100} is the position of the (100) peak, d_{002} and d_{100} are the interlayer distances calculated according to peak position of corresponding planes, ρ_{struc} is the structural density of carbon samples, SLD_C is the contrast of the scattering length density between the carbon matrix and the pores, ϕ_{pores} the volume fraction of the nanometer sized pores, ρ_{sample} is the samples density of studied non-graphitizable carbons and SLD_S represents the scattering length density of the powder grains in respect so surrounding vacuum.

Sample	q_{002} [nm^{-1}]	q_{100} [nm^{-1}]	d_{002} [nm]	d_{100} [nm]	ρ_{struc} [g cm^{-3}]	SLD_C [10^{10} cm^{-2}]	ϕ_{pores} [%]	ρ_{sample} [g cm^{-3}]	SLD_S [10^{10} cm^{-2}]
graphite	18.722	29.471	0.335	0.213	2.26	19.2	0	2.26	19.2
Corn@900°C	16.869	30.346	0.378	0.206	2.14	18.3	6.1	2.01	17.1
Corn@1200°C	16.709	30.287	0.376	0.207	2.13	18.1	6.3	1.99	16.9
Corn@1400°C	16.849	30.320	0.373	0.207	2.15	18.3	6.7	2.00	17.0
Corn@1600°C	17.089	30.323	0.368	0.207	2.18	18.5	8.6	1.99	16.9

Table S3: Structural parameters deduced from the component I_{mp}, where ΔSLD_C is the contrast of the scattering length density between the carbon matrix and the pores, ξ the correlation length beyond which the order is lost, f_a the disorder parameter, S_{mp} the micropore surface area, ϕ_{pores} the volume fraction of the nanometer sized pores, w_p the average pore width and w_c the average width of the carbon matrix.

Sample	$(\Delta SLD_c)^2$ [10^{20} cm^{-4}]	ξ [nm]	f_a [/]	S_{mp} [m^2 g^{-1}]	ϕ_{pores} [%]	W_p [nm]	w_c [nm]
Corn@900°C	331.9	0.306	0.73	351	6.1	0.33	4.97
Corn@1200°C	326.8	0.348	0.93	341	6.3	0.37	5.52
Corn@1400°C	333.7	0.423	0.52	295	6.7	0.45	6.31
Corn@1600°C	343.3	0.474	0.43	334	8.6	0.52	5.49

Table S4: Structural parameters deduced from the component I_{WAXS}, where w_G is the Gaussian FWHM, w_L is the Lorentzian FWHM, ξ the distortion length (extracted from FWHM) beyond which the long-range order is lost, R the length below which the layers can be considered as effectively flat and Σ the distortion length which determines the layer-layer distance above which the long range order is lost.

Sample	w_G [10^{-5} nm^{-1}]	w_L [nm^{-1}]	$\xi = 2/w_L$ [nm]	R [nm]	Σ [nm]
Corn@900°C	1	9.6	0.21	0.009	0.040
Corn@1200°C	2	9.5	0.21	0.012	0.030
Corn@1400°C	237	7.9	0.25	0.013	0.0687
Corn@1600°C	15	6.9	0.29	0.023	0.066

Note 1. Raman spectroscopy

Raman spectroscopy is a powerful tool to investigate the concentration of defects of the carbon materials. Two peaks may be observed (Figure S5a), namely the D band (1350 cm^{-1}) and the G band (1600 cm^{-1}). For Corn@1600°C there is also a 2D peak present at 2670 cm^{-1}, indicating the presence of few layer graphene-like structures. Concentration of defects was determined as the ratio of the integrated areas of the D and G band peaks. The values are presented in Table 1. The concentration of defects (I_D/I_G ratio) decreases with the increasing temperature of carbonization, indicating the graphitization of the material to some extent (Figure S5b). These results are in a good agreement with the data obtained by XRD. However, the I_D/I_G ratio is used to determine the concentration of defects in graphitic materials and is less accurate for non-graphitizable carbons.[13]

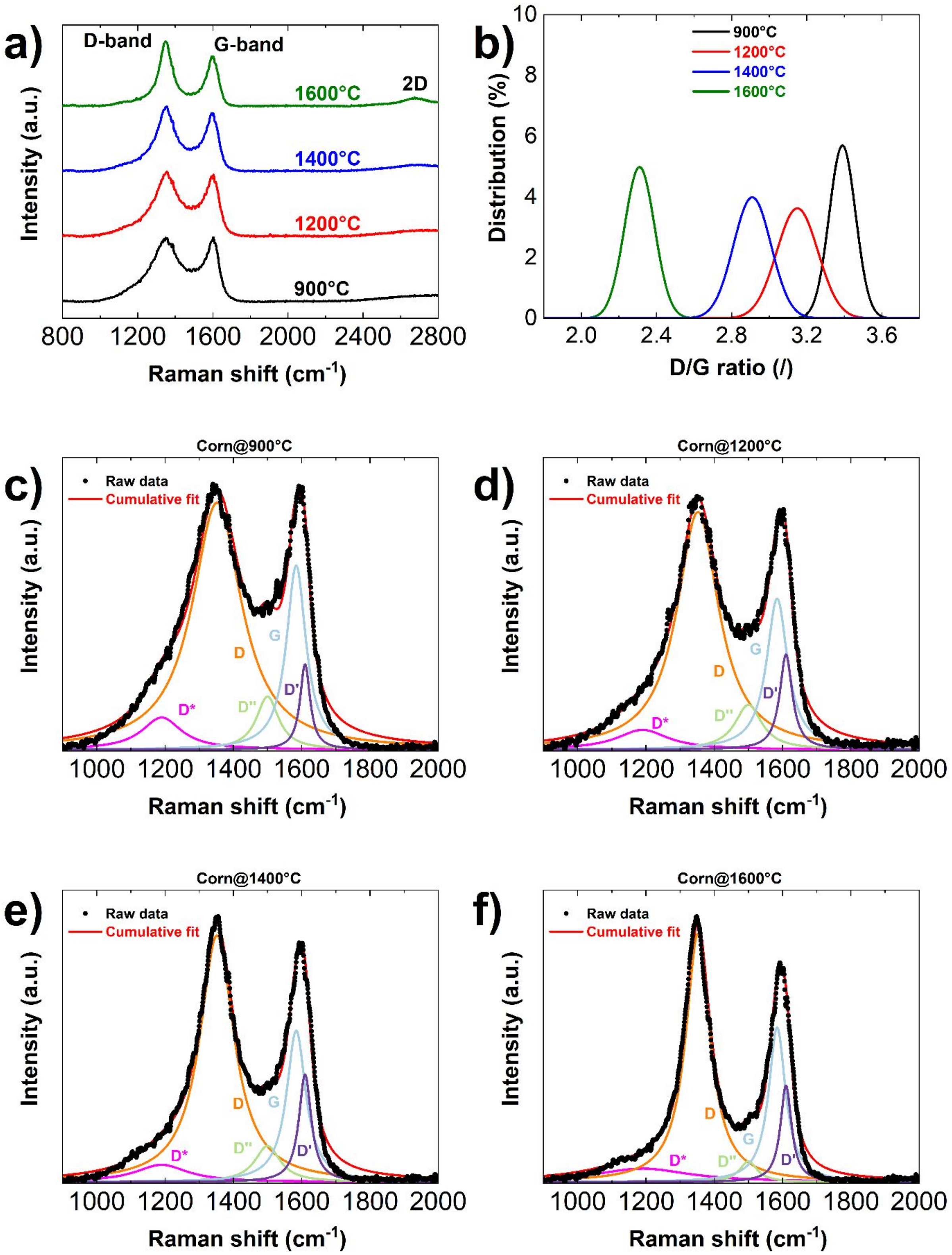

Figure S5: a) comparison of Raman spectra of corncob derived non-graphitizable carbon prepared at different temperatures, b) D/G ratio distribution for corncob derived non-graphitizable carbons prepared at different temperatures of carbonization, c-f) Raman spectra with corresponding fits and individual peaks present.

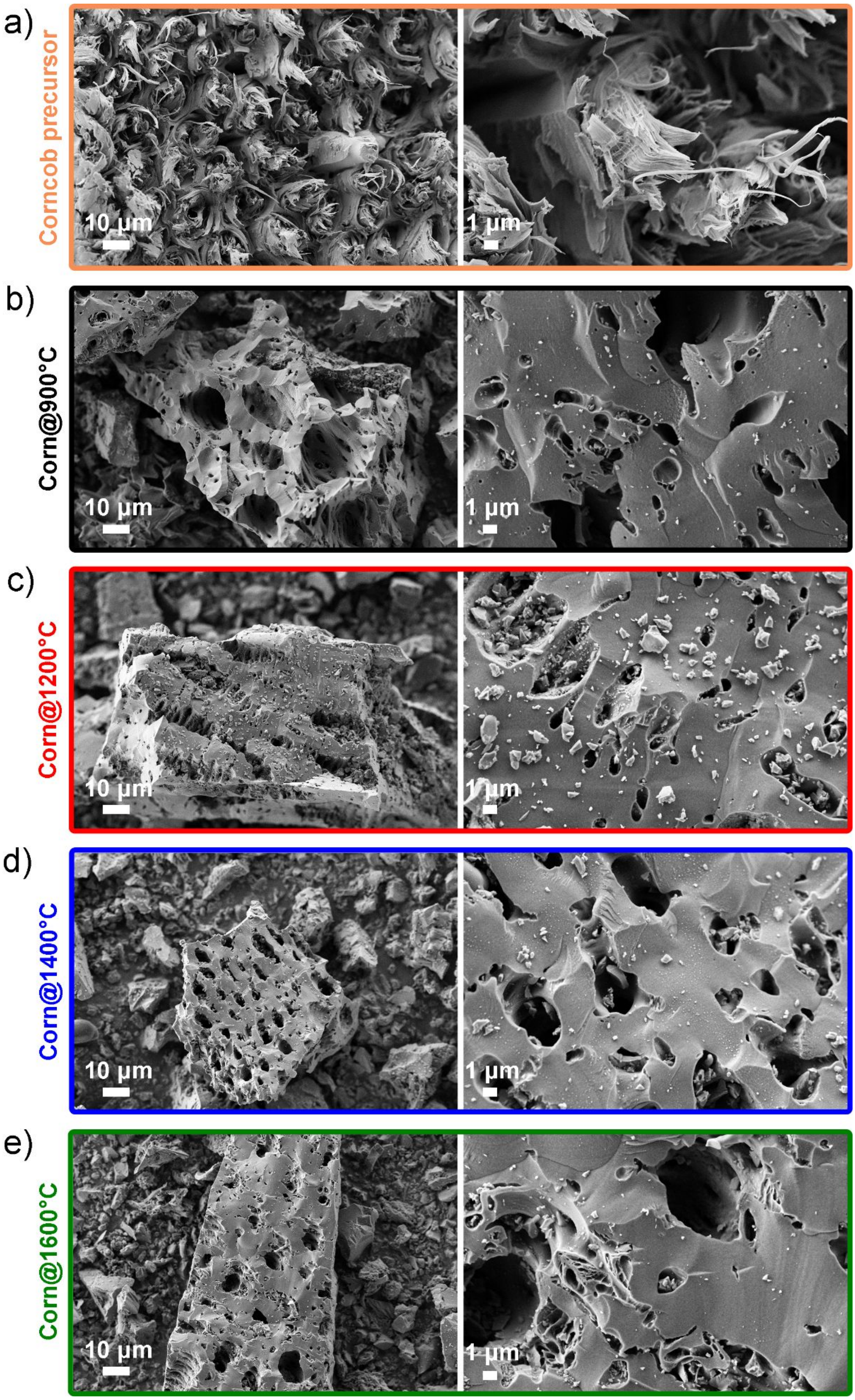

Figure S6: SEM images of corncob derived non-graphitizable carbons prepared at different temperatures of carbonization

a)

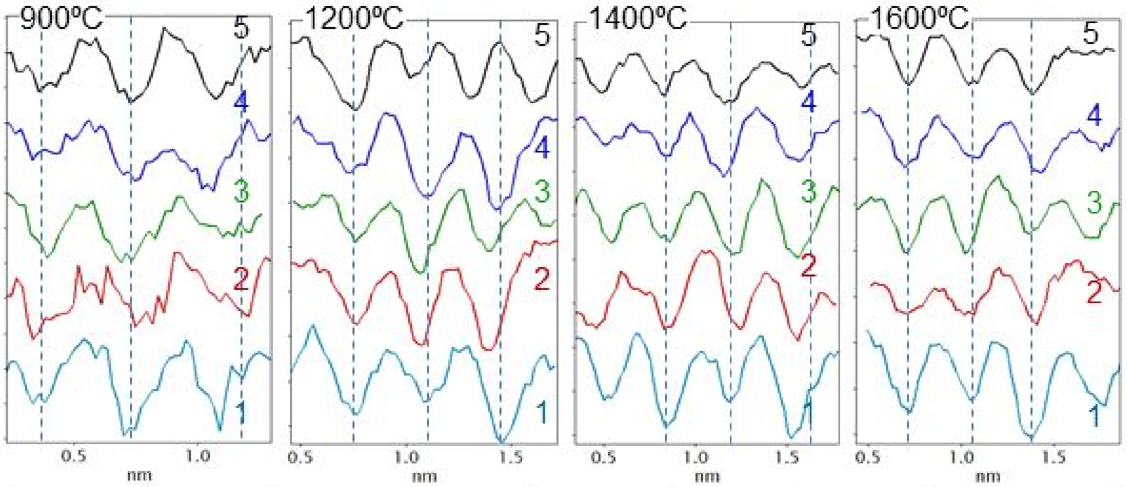

d, Å	Point 1	Point 2	Point 3	Point 4	Point 5	XRD, Å	Average, Å
**900ºC**	2.98	4.35	3.67	3.39	3.55	**3.78**	/
**1200ºC**	3.9	3.2	2.97	3.43	2.89	**3.76**	**3.28** ± 0.36
**1400ºC**	3.44	3.55	3.36	3.55	3.67	**3.73**	**3.51** ± 0.11
**1600ºC**	3.31	3.43	3.27	3.54	3.31	**3.68**	**3.37** ± 0.1

b)

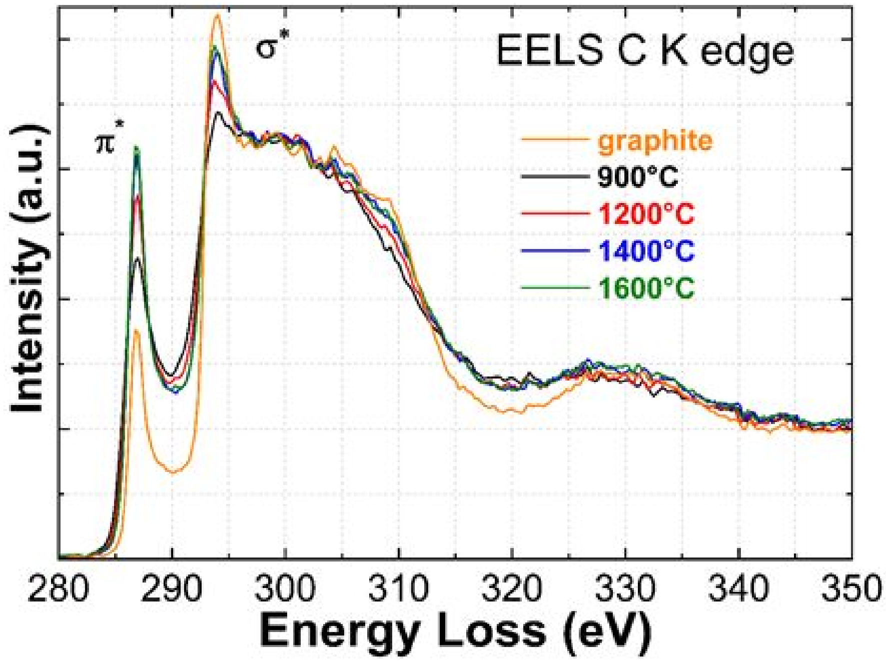

Figure S7: a) Intensity line profiles of Corn@900 ℃, Corn@1200 ℃, Corn@1400 ℃, and Corn@1600 ℃ samples, measured at the positions of white dashed lines drawn on the STEM-BF micrographs in the Figure 3 a-d. The pixel size at the given STEM magnification was 0.2 Å. The errors are given in 1σ. b) Normalized EELS C K edge spectra taken from all 4 samples as well as from pure graphite reference

Table S5: sp^2/sp^3 ratios and relative sp^2 amounts of corncob derived non-graphitizable carbons determined by EELS. For the purpose of comparison the sp^2/sp^3 ratio of Corn@1600°C was set to be 100%.

	sp^2/sp^3 ratio [/]	Relative sp^2 amount [%]
Corn@900°C	4.12	77
Corn@1200°C	4.71	88
Corn@1400°C	5.17	97
Corn@1600°C	5.36	100

*Table S6: Initial Coulombic efficiency (i*CE*) values of corncob derived non-graphitizable carbons.*

	iCE ($NaPF_6$/PC:EC) [%]
Corn@900°C	69.4
Corn@1200°C	77.8
Corn@1400°C	75.9
Corn@1600°C	66.1

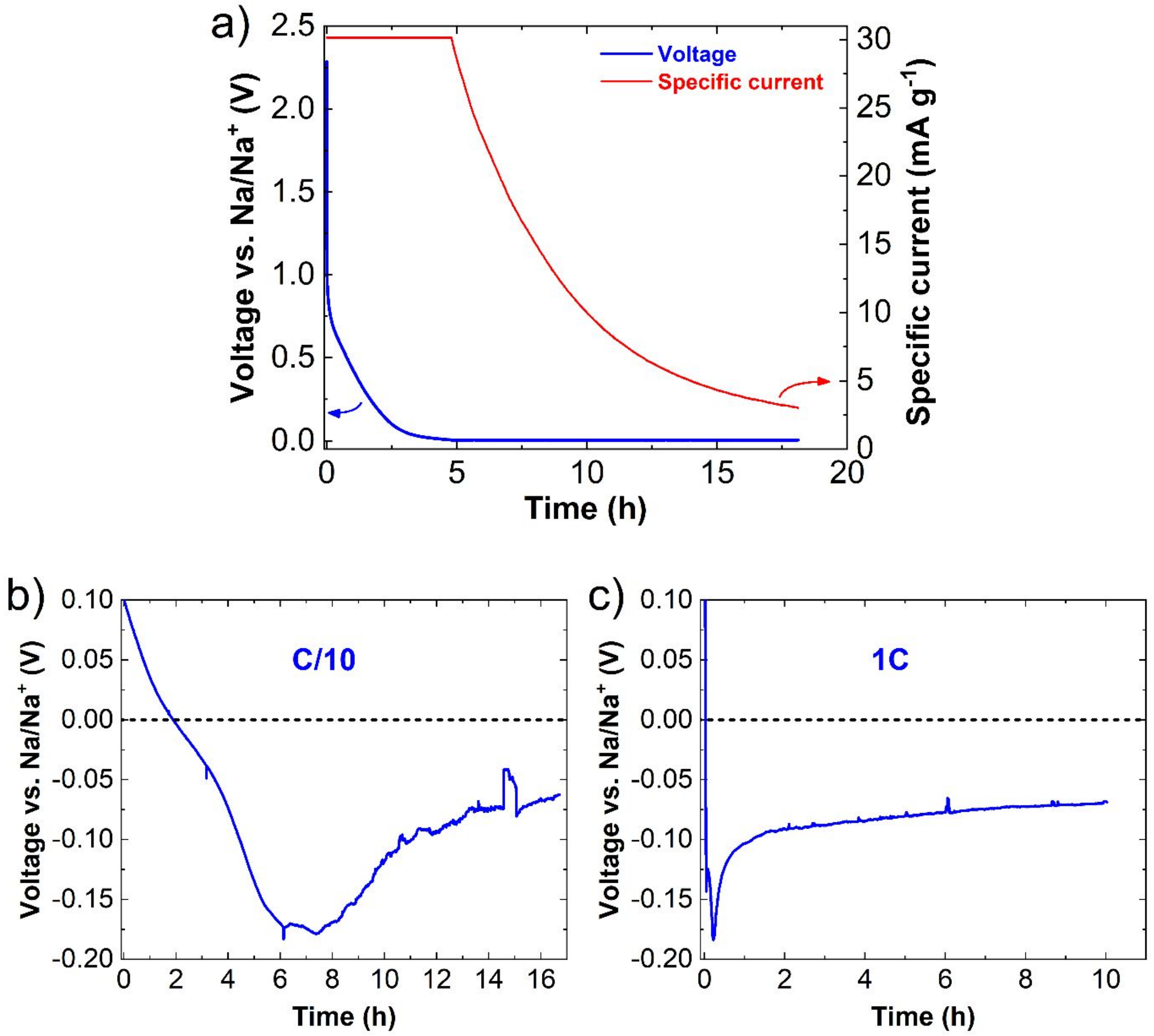

Figure S8: a) First cycle voltage and current profile of Corn@1400°C. Voltage profile of Corn@1400°C when sodiated to negative potentials at a b) C/10 current density and c) 1C current density.

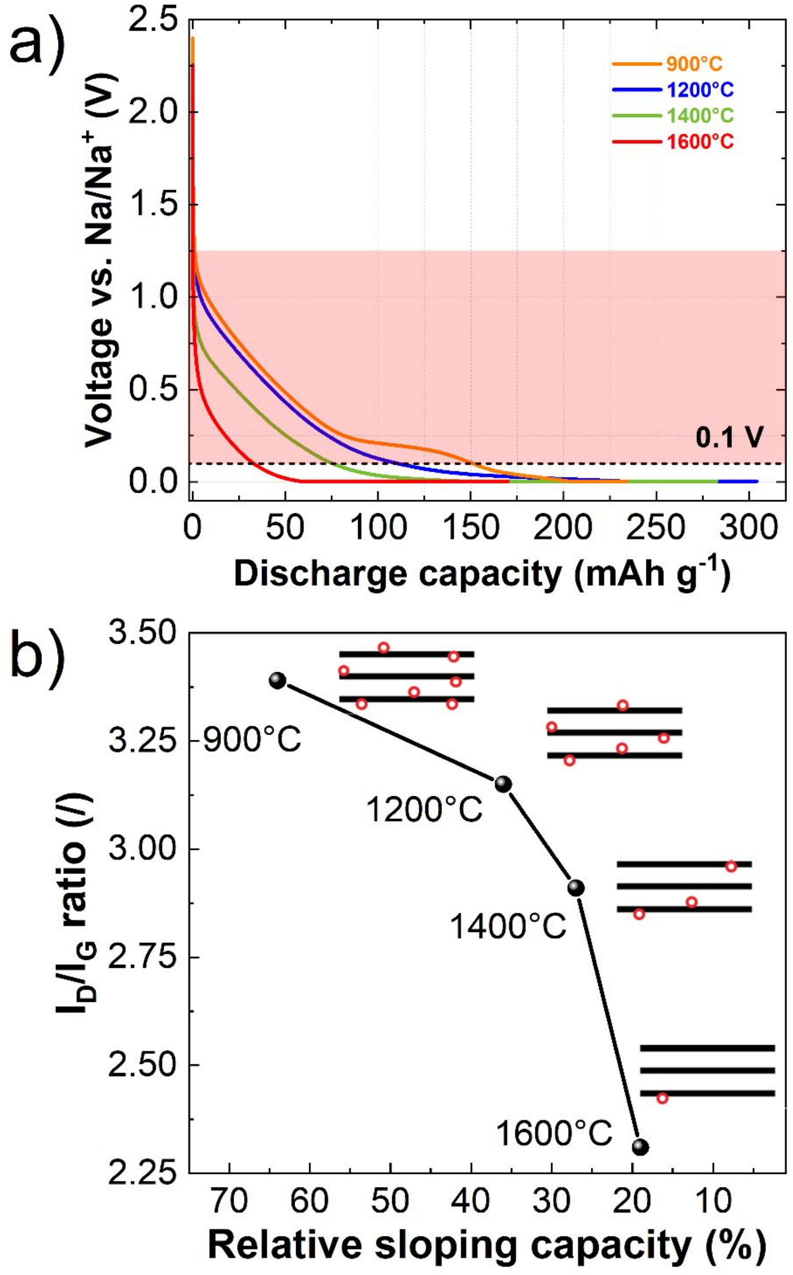

Figure S9: Correlation between a) galvanostatic curves and b) the I_D/I_G ratio and the relative sloping capacity of corncob derived non-graphitizable carbons at different temperatures of carbonization. The relative sloping capacities were calculated according to the second discharge as presented in Figure 4b in the main article.

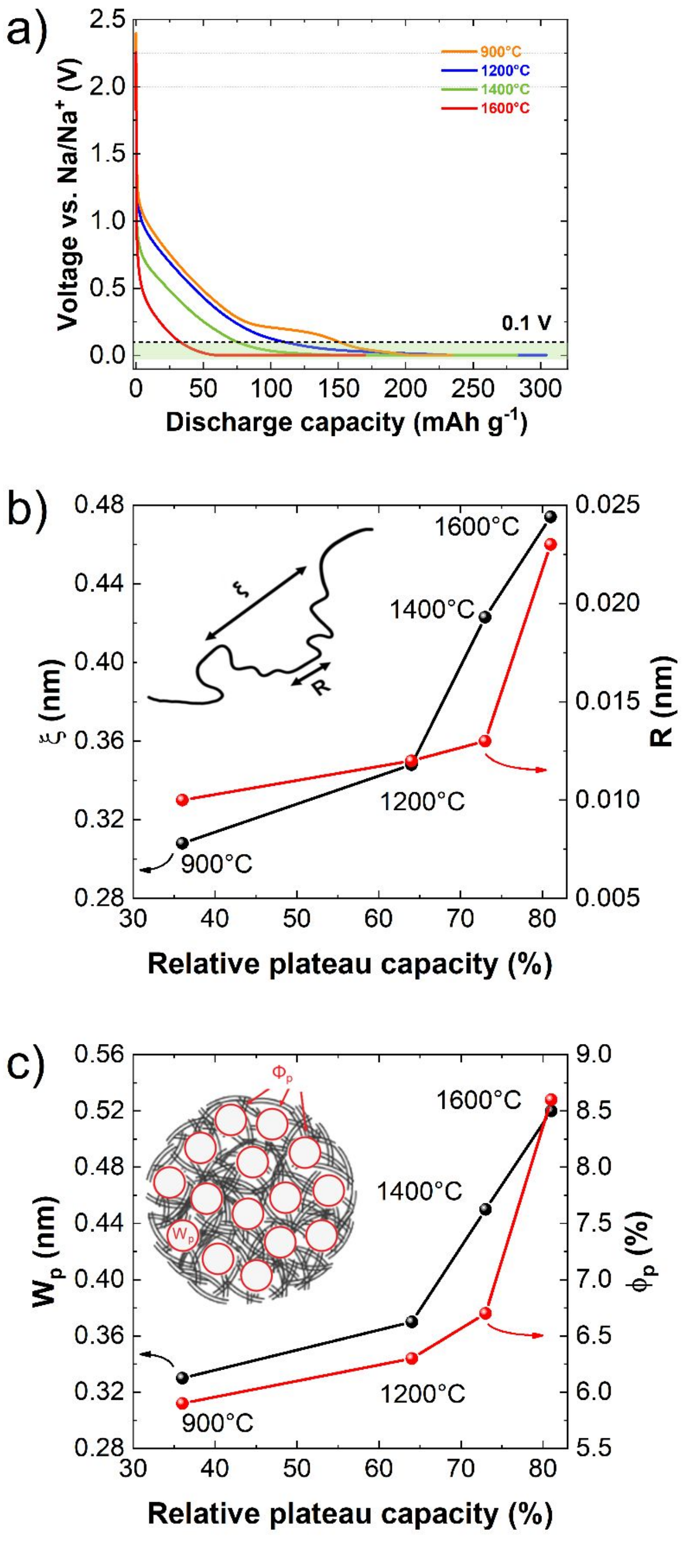

Figure S10: Correlation between the a) galvanostatic curves and b) correlation length describing the long-range order (ξ) and locally flat sections of the layers (R) with the relative plateau capacity and c) average pore width (W_p) and pore volume fraction (Φ_p) with the relative plateau capacity of corncob derived non-graphitizable carbons at different temperatures of carbonization. The relative plateau capacities were calculated according to the second discharge as presented in Figure 4b in the main article.

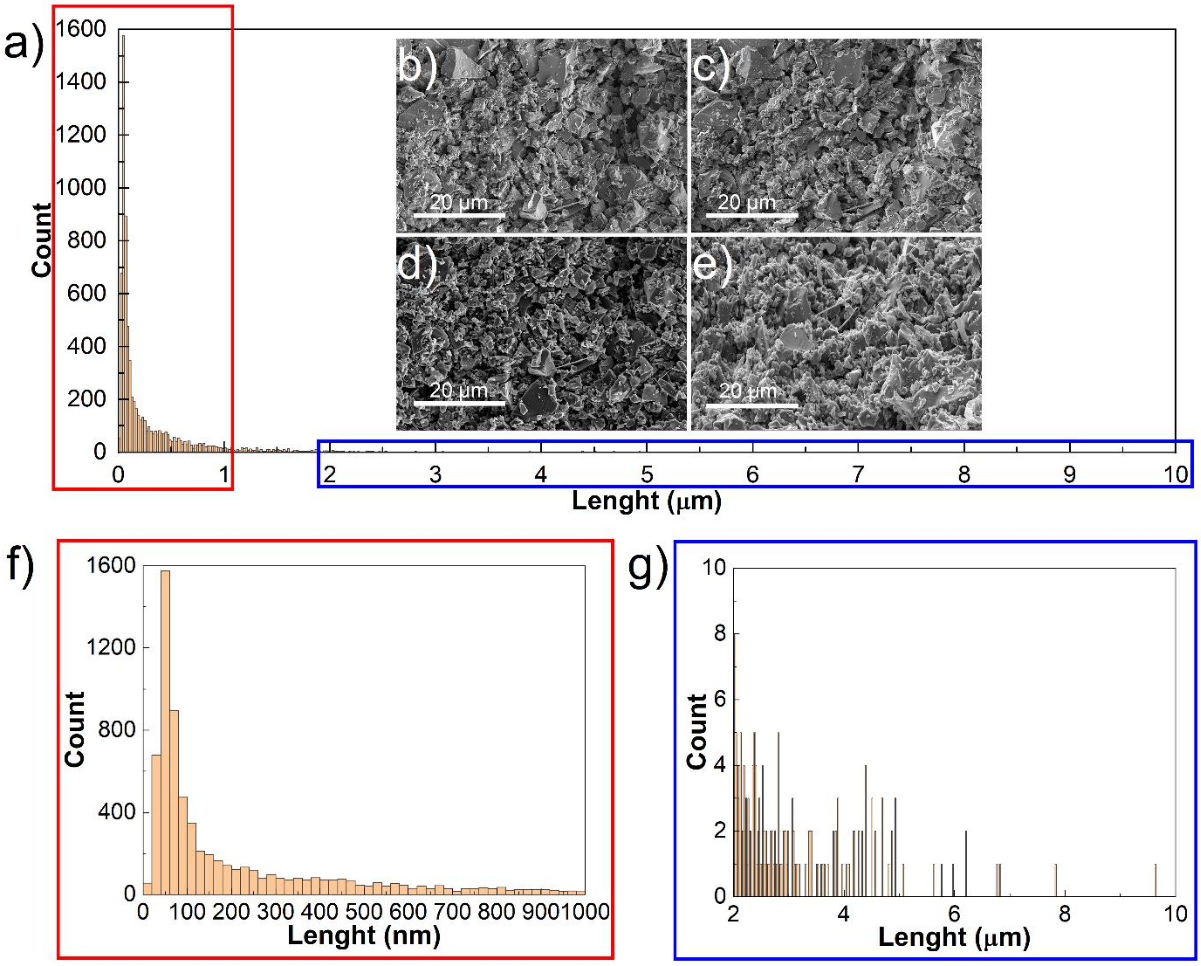

Figure S11: a) Statistical analysis of a particle size distribution from SEM images containing 13713 particles on a 2900 µm² surface area. SEM images of the same location with different detectors b) ETD detector and c) ICE detector at 2.00 kV. SEM images of two different location with d) ETD detector at 5.00 kV and e) ICE detector at 5.00 kV. f) Magnification of the statistical analysis of a particle size distribution from 0 to 1000 nm and g) Magnification of the statistical analysis of a particle size distribution from 2 to 10 µm.

Note 2. Surface analysis of electrodes by FIB-SEM

Prior FIB cross-section fabrication, sample surface was initially protected with 300 nm platinum (Pt) layer by "in situ" inducing Pt-organometallic gas precursor with electron beam (2kV @ 0.40 nA). Subsequently, Pt layer thickness was increased up to 1 µm by "in situ" inducing Pt gas precursor with Ga+ ion beam (30 kV @ 0.23 nA). Cross-sections were made using focused Ga^{+} ions at 30 kV @ 9.30 nA with sequential reducing currents down to 0.40 nA for the case of final ion polishing step. Detailed morphological information and phase contrast images were obtained by using in-column TLD detector (SE and BSE mode) at low energy pre-monochromated electron beam (1 kV @ 50 pA, UHR, U-mode).

EDX elemental distribution analysis

The elemental composition of the pristine electrode, surface and cross-section is presented in Figure S10. The EDX mapping shows a homogenious distribution of the carbon and fluorine from the binder. The EDX of the surface has the similar chemical composition as the cross-sectional EDX (Figures S10a and S10b)

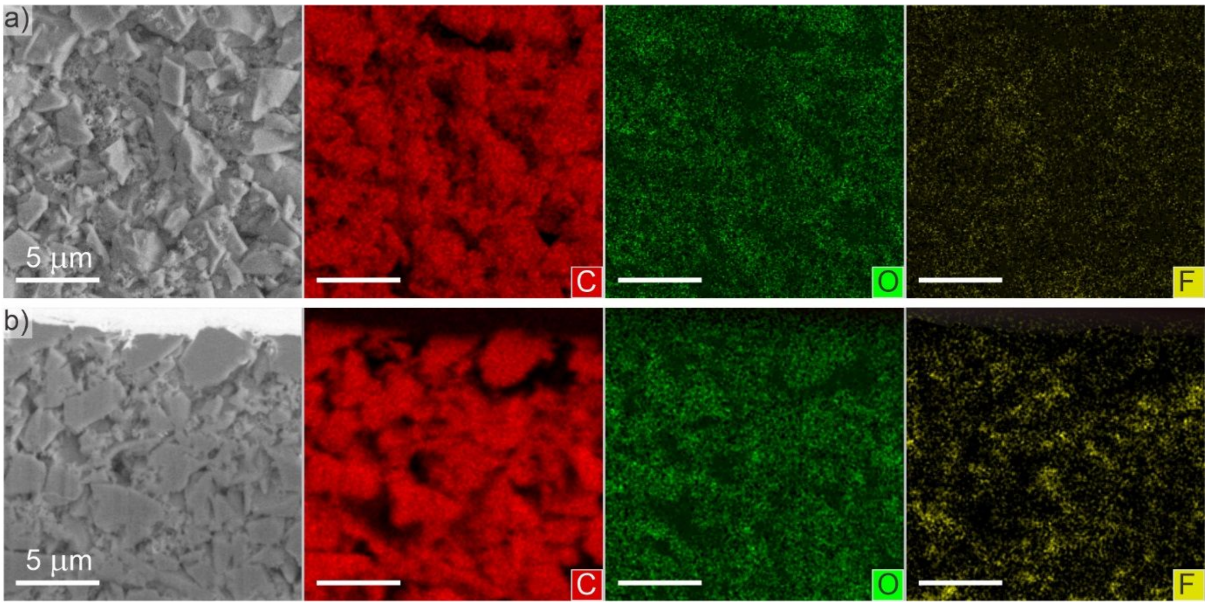

Figure S12: Surface (a) and cross-sectional FIB-SEM analysis (b) with the corresponding EDX elemental distribution maps of the pristine sample

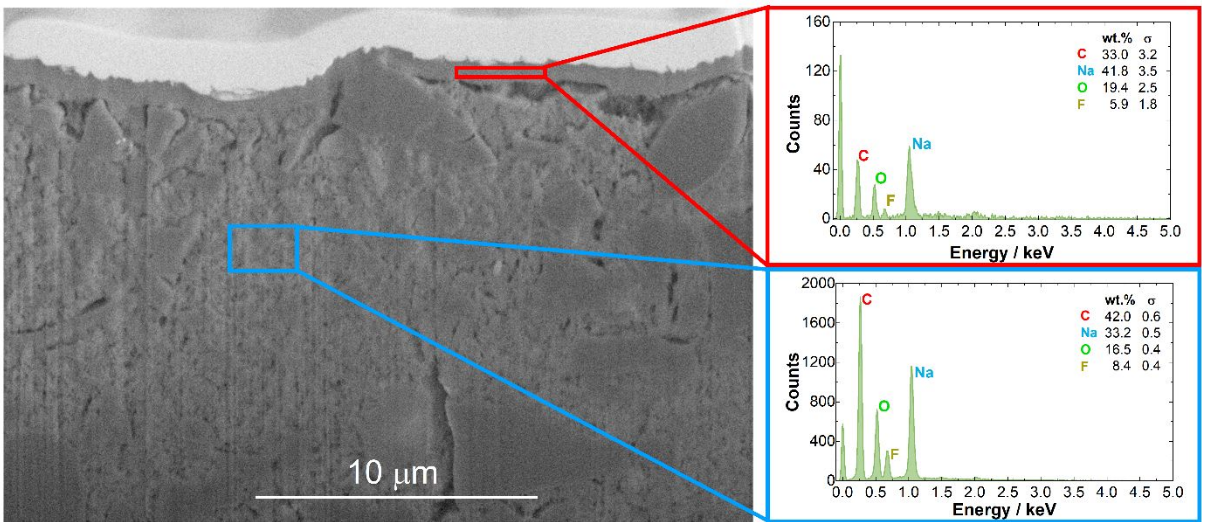

Figure S13: Quantitative EDX spectra localized on SEI layer (red) and representative area inside the electrode (blue)

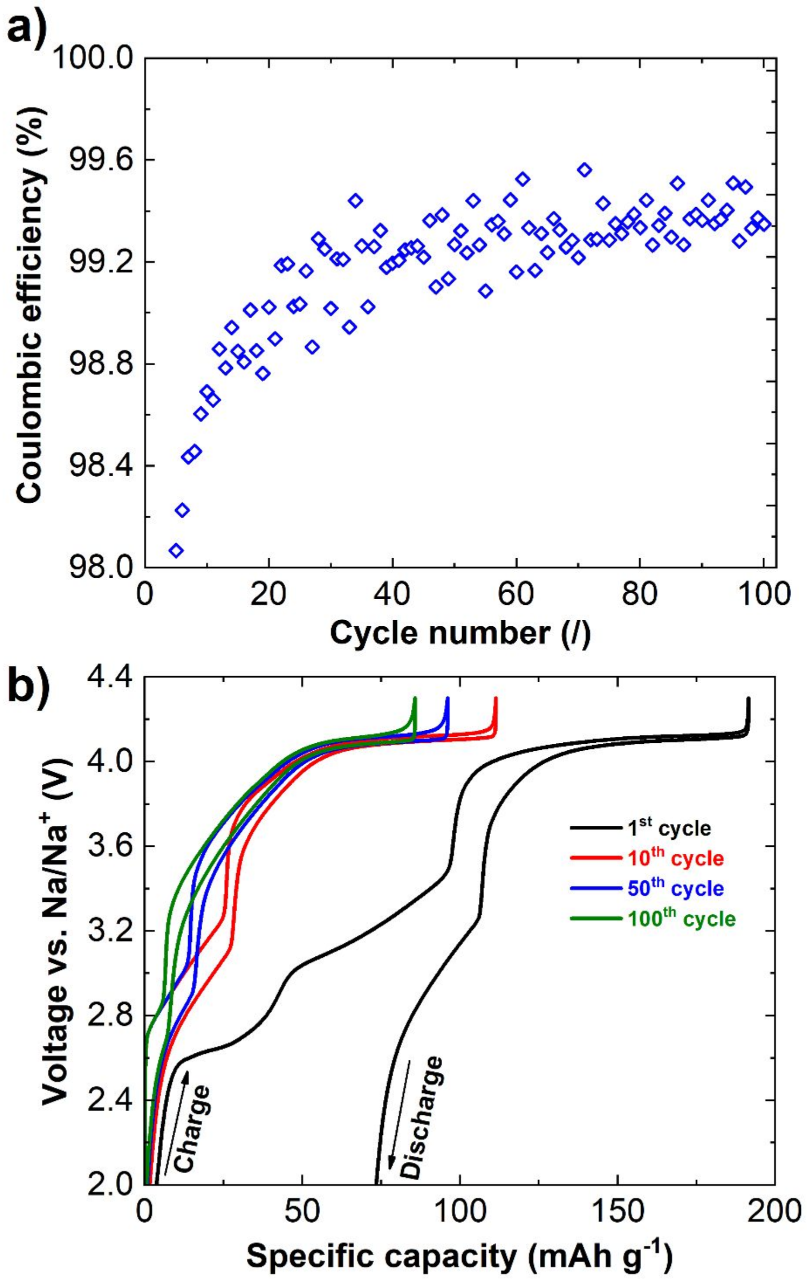

Figure S14: a) Zoom in of Coulombic efficiency for the NVPF/Corn@1400°C two-electrode cell, b) electrochemical curves representing the 1st, 10th, 50th and 100th cycle of NVPF/Corn@1400°C two-electrode cell.

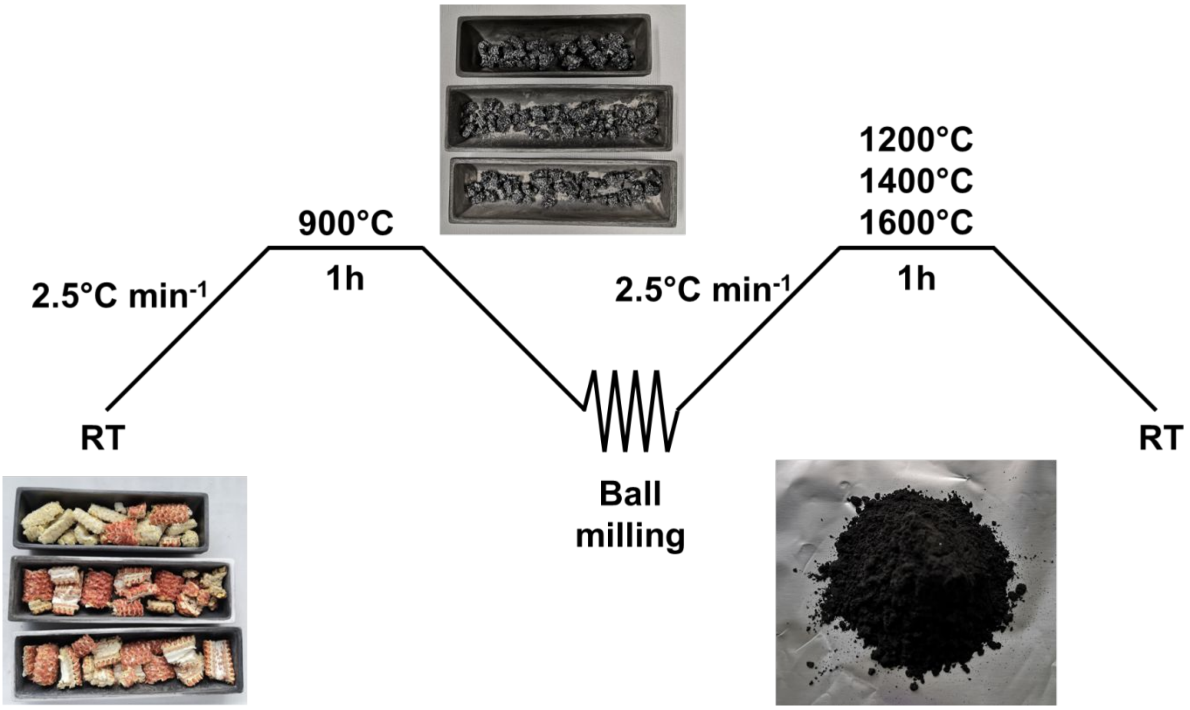

Figure S15 Heat treatment process flow rate.

References

(1) Bragg, W. H.; Bragg, W. L. The Reflection of X-Rays by Crystals. *Proc. R. Soc. London. Ser. A, Contain. Pap. a Math. Phys. Character* **1913**, *88* (605), 428–438..

(2) B.E.Warren. *X-Ray Diffraction*; DoverPublications Inc.: New York, 1990.

(3) Dou, X.; Hasa, I.; Hekmatfar, M.; Diemant, T.; Behm, R. J.; Buchholz, D.; Passerini, S. Pectin, Hemicellulose, or Lignin? Impact of the Biowaste Source on the Performance of Hard Carbons for Sodium-Ion Batteries. *ChemSusChem* **2017**, *10*, 2668–2676.

(4) Lake, J. An Iterative Method of Slit-Correcting Small Angle X-Ray Data. *Acta Crystallogr.* **1967**, *23*, 191–194.

(5) Pohmann, R. Physical Basics of NMR. In *In vivo NMR Imaging. Methods in Molecular Biology*, vol 771. Humana Press.

(6) Saurel, D.; Segalini, J.; Jauregui, M.; Pendashteh, A.; Daffos, B.; Simon, P.; Casas-Cabanas, M. A SAXS Outlook on Disordered Carbonaceous Materials for Electrochemical Energy Storage. *Energy Storage Mater.* **2019**, *21*, 162–173.

(7) Schubert, K.-V.; Strey, R.; Kline, S. R.; Kaler, E. W. Small Angle Neutron Scattering near Lifshitz Lines - Transition from Weakly Structured Mixtures to Microemulsions. *J. Chem. Phys.* **1994**, *101* (6), 5343–5355.

(8) Teubner, M.; Strey, R. Origin of the Scattering Peak in Microemulsions. *J. Chem. Phys.* **1987**, *87* (5), 3195–3200.

(9) Porod, G. General Theory. In *Small Angle X-Ray Scattering*; Glatter, O., Kratky, O., Eds.; Academic Press Inc. London Ltd: London, **1983**; 17–51.

(10) Vonk, C. G. The Small-Angle Scattering of Distorted Lamellar Structures. *J. Appl. Crystallogr.* **1978**, *11*, 541–546.

(11) Saurel, D.; Ségalini, J.; Jáuregui, M.; Pendashteh, A.; Daffos, B.; Simon, P.; Casas-Cabanas, M. Corrigendum to "A SAXS Outlook on Disordered Carbonaceous Materials for Electrochemical Energy Storage" [Energy Storage Mater. 21 (2019) 162–173]. *Energy Storage Mater.* **2020**, *28*, 418.

(12) NIST Neutron activation and scattering calculator https://www.ncnr.nist.gov/resources/activation/ (accessed May 28, 2021).

(13) Claramunt, S.; Varea, A.; López-Díaz, D.; Velázquez, M. M.; Cornet, A.; Cirera, A. The Importance of Interbands on the Interpretation of the Raman Spectrum of Graphene Oxide. *J. Phys. Chem. C* **2015**, *119* (18), 10123–10129.
